# Wordless intervention for people with epilepsy and learning disabilities (WIELD): a randomised controlled feasibility trial

**DOI:** 10.1136/bmjopen-2016-012993

**Published:** 2016-11-10

**Authors:** Silvana E Mengoni, Bob Gates, Georgina Parkes, David Wellsted, Garry Barton, Howard Ring, Mary Ellen Khoo, Deela Monji-Patel, Karin Friedli, Asif Zia, Lisa Irvine, Marie-Anne Durand

**Affiliations:** 1 Department of Psychology, Centre for Health Services and Clinical Research, University of Hertfordshire, Hatfield, UK; 2 Institute for Practice, Interdisciplinary Research and Enterprise (INSPIRE), University of West London, London, UK; 3 Learning Disabilities Services, Hertfordshire Partnership University NHS Foundation Trust, St Albans, UK; 4 Norwich Medical School and Norwich Clinical Trials Unit, University of East Anglia, Norwich, UK; 5 Department of Psychiatry, University of Cambridge, School of Clinical Medicine, Cambridge, UK; 6 Research and Development, Hertfordshire Partnership University NHS Foundation Trust, St Albans, UK; 7 Division 4, Mental Health, NIHR Clinical Research Network: Eastern, UK; 8 Centre for Research in Primary and Community Care, University of Hertfordshire, Hatfield, UK; 9 The Dartmouth Institute for Health Policy and Clinical Practice, Dartmouth College, Hanover, New Hampshire, USA

**Keywords:** PSYCHIATRY, MENTAL HEALTH

## Abstract

**Objective:**

To investigate the feasibility of a full-scale randomised controlled trial of a picture booklet to improve quality of life for people with epilepsy and learning disabilities.

**Trial design:**

A randomised controlled feasibility trial. Randomisation was not blinded and was conducted using a centralised secure database and a blocked 1:1 allocation ratio.

**Setting:**

Epilepsy clinics in 1 English National Health Service (NHS) Trust.

**Participants:**

Patients with learning disabilities and epilepsy who had: a seizure within the past 12 months, meaningful communication and a carer with sufficient proficiency in English.

**Intervention:**

Participants in the intervention group used a picture booklet with a trained researcher, and a carer present. These participants kept the booklet, and were asked to use it at least twice more over 20 weeks. The control group received treatment as usual, and were provided with a booklet at the end of the study.

**Outcome measures:**

7 feasibility criteria were used relating to recruitment, data collection, attrition, potential effect on epilepsy-related quality of life (Epilepsy and Learning Disabilities Quality of Life Scale, ELDQOL) at 4-week, 12-week and 20-week follow-ups, feasibility of methodology, acceptability of the intervention and potential to calculate cost-effectiveness.

**Outcome:**

The recruitment rate of eligible patients was 34% and the target of 40 participants was reached. There was minimal missing data and attrition. An intention-to-treat analysis was performed; data from the outcome measures suggest a benefit from the intervention on the ELDQOL behaviour and mood subscales at 4 and 20 weeks follow-up. The booklet and study methods were positively received, and no adverse events were reported. There was a positive indication of the potential for a cost-effectiveness analysis.

**Conclusions:**

All feasibility criteria were fully or partially met, therefore confirming feasibility of a definitive trial.

**Trial registration number:**

ISRCTN80067039.

**Strengths and limitations of this study**

- This feasibility study is the first completed randomised controlled trial addressing the unmet information and self-management needs of people with learning disabilities and epilepsy.
- Effective collaboration with local clinical services and patient and public representatives facilitated recruitment to target.
- A mixed-methods design clearly addressed the feasibility criteria and highlighted improvements for a definitive trial through quantitative and qualitative data.
- Despite efforts to recruit people with severe learning disabilities, this population are under-represented in the study.
- There were important variations in the extent that carers involved participants in the questionnaires, and how the booklet was used, which cannot be examined in a feasibility study.

## Introduction

People with learning disabilities (∼1.5 million people in the UK) face an increased risk of health problems, poorer outcomes than in the general population and premature, avoidable deaths.^1–3^ Epilepsy is the most common neurological condition for people with learning disabilities^4^ with a prevalence rate of 22%,^5^ compared with 0.4–1% of the general population, which increases with the severity of the learning disability.^6^

Epilepsy in people with learning disabilities typically begins in childhood, and has a complex presentation, with people often experiencing frequent seizures, generalised seizures and multiple seizure types.^7^ Poorly controlled and severe seizures are more likely than in the general population with ∼70% of people with learning disabilities and epilepsy continuing to have seizures despite taking antiepileptic medication.^7^ ^8^ Comorbid health and mental health issues as well as cognitive impairment can make the management of epilepsy difficult.^9^ This can have a negative impact on care needs, daily activities, work, quality of life, instances of hospitalisation, mortality and health and social care costs.^10–15^

Guidance from the National Institute for Health and Care Excellence (NICE) in England states that people with learning disabilities and epilepsy should be offered the same services, investigations and care as the general population, as well as information and support that is tailored to their needs in order to promote autonomy and empowerment.^16^ However, this group continue to experience poor access to diagnostic investigations and specialist services; access to services with appropriate expertise appears to be highly dependent on location.^7^ ^15^ ^17^ ^18^

People with learning disabilities are often excluded from clinical trials^19^ resulting in a weak evidence base for effective treatment and services for this population. This is particularly evident in epilepsy research,^12^ illustrated by a recent review of service responses for epilepsy in learning disabilities which identified no evaluations using randomisation or a matched comparison group.^15^ Despite calls for non-pharmacological interventions to be used alongside antiepileptic medication,^20^ there is little evidence for the effectiveness of such interventions due to the lack of robust trials in this area.^21^

People with learning disabilities and epilepsy often lack appropriate skills training to manage their epilepsy, as well as provision of accessible information.^12^ ^18^ There is also a need to improve communication between healthcare professionals and people with learning disabilities regarding decision-making about epilepsy management.^12^ In the general population, self-management interventions have been found to improve people's understanding and management of epilepsy along with their tolerability and adherence to medication.^22–24^ In contrast, self-management interventions are rarely implemented or evaluated with people with learning disabilities and epilepsy.^15^ ^25^ Such interventions have potential to improve knowledge and epilepsy management, with a likely improvement in quality of life.

‘Getting on With Epilepsy’ is a picture booklet designed for people with learning disabilities and epilepsy,^26^ and is part of the Beyond Words series of booklets.^27^ This booklet tells the story of a young adult with epilepsy, and promotes understanding of epilepsy, self-management and empowerment. The booklet uses pictures rather than words to tell a story. It is suitable for people with a range of communication styles and capitalises on the visual literacy and pictorial superiority of people with learning disabilities, who tend to process and assimilate pictorial information better than textual information.^28–30^ The range of Beyond Words booklets are popular and have received awards, but have not, as yet, been evaluated in a controlled study, nor routinely adopted in the health or social care sector in the UK. The epilepsy picture booklet has the potential to be an inexpensive and simple way of addressing the information and self-management needs of people with learning disabilities and epilepsy.

The objectives of the Wordless Intervention for Epilepsy in Learning Disabilities (WIELD) randomised controlled feasibility trial (feasibility RCT) were to:

1. Assess the feasibility of undertaking a RCT of the picture booklet as an intervention for epilepsy in people with learning disabilities.
2. Explore the feasibility of collecting resource use and quality of life data so as to inform the design of the health economics component of a future definitive trial.
3. Assess the study procedures' and intervention's acceptability among adults with learning disabilities, carers and health professionals.

The full protocol for this study has previously been published.^31^

## Methods

### Design and setting

The study had a two-arm, single-centre parallel design, and was conducted at one National Health Service (NHS) Trust in England where participants were Hertfordshire Partnership University NHS Foundation Trust (HPFT) patients over a 20-month period from July 2014 to February 2016. Participants were recruited through seven epilepsy clinics, and then randomised to the intervention or control group.

### Participants

Eligible patients were 18 years of age or over, with a confirmed clinical diagnosis of a learning disability (IQ≤70) and epilepsy. Patients had to have experienced at least one seizure over the previous 12 months and have meaningful verbal or non-verbal communication which was operationalised as being able to tell or follow the story in the picture booklet. Originally, only patients with verbal communication were eligible. This was changed before the start of recruitment based on feedback from patient representatives, carers and health professionals. The carers of the patients had to be sufficiently proficient in English to read and complete the study questionnaires. Patients were excluded if they had a visual impairment or dementia, or if they had used the picture booklet within the past 12 months.

A subgroup of participants and their carers were invited to take part in a semistructured interview after completion of the final follow-up assessment to explore feasibility and acceptability of the study and intervention. Random sampling was originally proposed, but this was modified to purposeful sampling to ensure a range of views and backgrounds were represented. A sample of health professionals were also invited to participate in a semistructured interview.

### Consent

Eligible patients were identified by epilepsy nurses and consultant psychiatrists who ran epilepsy clinics at HPFT. A log of eligible patients was created using the inclusion and exclusion criteria. These patients were then contacted to invite them to take part in the study through a postal invitation pack containing standard and easy-read information (see online supplementary material for easy-read information sheet). This was then followed up with a phone call ∼1 week later. A researcher subsequently visited interested patients and carers to discuss the study. If the patient had capacity to decide whether to participate, they completed an easy-read consent form. If the carer felt that the patient did not have capacity, a consultee declaration form was used. Originally the visit was stated to occur at an epilepsy clinic but this was changed, before recruitment started, to allow patients and carers to choose to be seen at their home if that was convenient. The original proposal also stated that consenting, randomisation, data collection and the intervention would all be conducted by a researcher with a research nurse background. In order to maximise recruitment, this was changed during the study to allow a trained clinical studies officer to also take on this role.

10.1136/bmjopen-2016-012993.supp1 Supplementary easy-read information sheet

### Randomisation and blinding

Participants were randomly allocated individually online by a researcher using a database on a secure website. The randomisation (1:1 allocation ratio) was ‘blocked’ into groups of six. Owing to the nature of the intervention, it was not possible for the participants or research team to be blind to group allocation.

### Intervention

The researchers who conducted the intervention received training from Beyond Words about how to use the booklet. Participants in the intervention condition, along with their carer, met the researcher at a convenient location. During this meeting, the ‘Getting on With Epilepsy’ booklet was introduced. This involved the participant going through the booklet at their own pace and being encouraged to talk about what they could see and how they felt, as appropriate.

As well as verbal guidance on how to use the booklet, the participant and carer were advised that there was further guidance and resources at the back of the booklet, and they were given further information produced by Beyond Words. Participants kept the booklet, and were asked to look at the booklet at least twice more at home over the 20-week study period. Participants or carers received a phone call ∼2 weeks later to discuss any issues they may have had with the booklet. The duration of all contact between researcher, participant and carer was noted by the research team, in order to estimate the cost of the intervention.

Participants in the control condition received routine information and services. At the end of the study, control group participants were provided with a copy of the booklet and information about how to use it.

### Outcome measures

The feasibility criteria were developed a priori to reflect key aspects of the study which would be necessary for a full-scale trial. The seven feasibility criteria were:

- Rate of eligible patients recruited is higher than 60%.
- At least 80% of study measures have been completed.
- Discontinuation rates fall under 20%.
- The likely location of the improvement in the primary outcome (quality of life) is at least 10%.
- Good feasibility and acceptability of the study methodology.
- Good acceptability of the intervention among participants, carers and health professionals.
- Collection of resource use and quality of life data is feasible, and the increase in treatment cost will be minimal.

**Figure 1:**
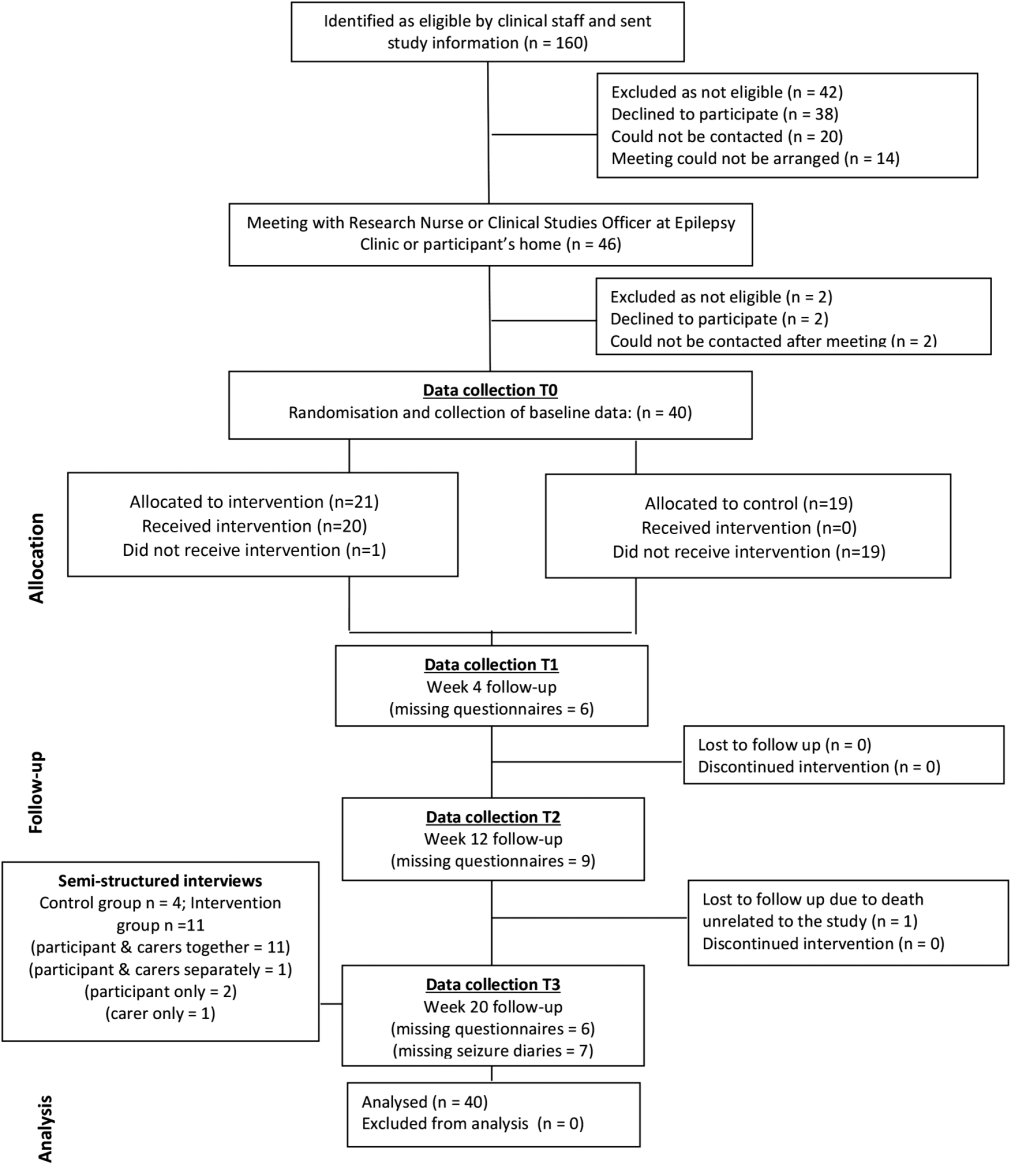
Study flow chart.

Each participant was assessed at baseline (T0), and at 4 (T1), 12 (T2) and 20 (T3) weeks after randomisation (figure 1) using the outcome measures detailed in table 1. The primary outcome measure was the Epilepsy and Learning Disabilities Quality of Life Scale (ELDQOL).^32^ ^33^ Seizure severity and control were secondary outcomes, as improved management of epilepsy has the potential to reduce seizures. Information was also collected about how often the intervention group participants used the booklet, how long for and who with. Participants were asked about any other epilepsy resources they had used, to explore whether booklet use led to further information-seeking.

**Table 1:** Study outcome measures

Outcome	Data collection method	T0 Baseline	T1 Week 4	T2 Week 12	T3 Week 20
Number of eligible patients and number recruited	Case report form	✓			
Discontinuation rates across both groups and reasons	Case report form	✓	✓	✓	✓
Demographic data	Questionnaire: context-specific questions about age, sex, ethnicity, and living circumstances of the participant, the type of care provided by the carer and their relationship to the participant	✓			
Patterns of use of the Beyond Words booklet (intervention group only)	Questionnaire: context-specific questions		✓	✓	✓
Use of other epilepsy-related information	Questionnaire: context-specific questions	✓			✓
Quality of life as the primary outcome measure	Questionnaire: ELDQOL scale, which consists of four subscales: behaviour, seizure severity, mood and side effects^32^ ^33^	✓	✓	✓	✓
Seizure severity as a secondary outcome measure	Questionnaire: ELDQOL seizure severity subscale	✓	✓	✓	✓
Seizure control as a secondary outcome measure	Seizure diary				✓
Health-related quality of life	Questionnaire: EQ-5D-5L index and visual analogue scale (EQ-VAS)—proxy version^34^	✓	✓	✓	✓
Health and social services and resources use	Questionnaire: context-specific resource use questions	✓			✓
Feasibility and acceptability of the study procedures	Semistructured interview				✓
Use and perceived usefulness of existing resources and services	Semistructured interview				✓
Information and self-management support needs	Semistructured interview				✓
Perceived acceptability of the intervention	Semistructured interview				✓
Perceived barriers and facilitators to the use and dissemination of the intervention in routine care	Semistructured interview				✓

The study questionnaires were designed to be completed by the carer with the participant's input, where possible. The baseline questionnaire was completed with a researcher present. The follow-up questionnaires were sent and returned by post.

The semistructured interview schedule for the health professionals and participants and carers aimed to explore the acceptability of the study design and intervention and perceived feasibility of using the booklet in a routine care setting. The interview schedule also explored the self-management, information and support needs of participants, in relation to their experience of epilepsy, and this is reported elsewhere.^35^

### Sample size

One of the study objectives was to assess variation in the ELDQOL in order to determine whether the intervention is likely to achieve a 10% increase in the participants' quality of life and to allow exclusion of an effect size of <0.^36^ Ten per cent equates to an effect size of ∼0.4. In a full-scale trial, this would require a total sample size of 174 (1**−**β=0.8, α=0.05), which indicates a sample size of 16 per group (eg, 9% per group) in the feasibility study. Assuming a conservative 25% drop out, the recruitment target was a sample size of 20 per group (40 participants in total). For the qualitative aspect of the study, we aimed to interview a subgroup of 15 participants and carers.

### Data analysis

The analysis aimed to determine the feasibility of conducting a full-scale RCT by addressing the objectives and feasibility criteria outlined in table 2.

**Table 2:** Objectives, feasibility criteria and key findings

Objectives	Feasibility criteria (if applicable)	Feasibility criterion met and suggested amendments if not met	Outcome
Evaluate the recruitment rate and number of eligible patients recruited (quantitative)	Feasibility criterion 1: rate of eligible patients recruited is higher than 60%	*Criterion not met overall* (although target recruitment was met) Amendments: Extend recruitment period Develop materials to increase recruitment of patients with severe learning disabilities	Target recruitment was met (n=40) Per cent of patients recruited from all those screened: 25% Per cent of patients recruited from eligible screened patients: 34%
Evaluate completion rates (quantitative)	Feasibility criterion 2: at least 80% of study measures have been completed	*Criterion met*	85% of all study measures were completed.
Measure discontinuation rates across both arms (quantitative)	Feasibility criterion 3: discontinuation rates fall under 20%	*Criterion met*	No discontinuations with the exception of one death (unrelated to the study)
Assess the variability of the primary outcome measure (quality of life) (quantitative)	Feasibility criterion 4: the likely location of the improvement in the primary outcome (quality of life) is at least 10%.	*Criterion partially met* Amendments: Omit medication subscale Develop an epilepsy knowledge measure	The 80% CI of the effect size excludes zero in favour of the intervention group on ELDQOL behaviour (T1; T3) and mood subscales (T1; T3). The 80% CI of the effect size includes the target effect size (10% improvement) for ELDQOL behaviour (T3).
Assess the feasibility and acceptability of the study methodology (qualitative)	Feasibility criterion 5: good feasibility and acceptability of the study methodology	*Criterion met*	Qualitative data indicated high acceptability and feasibility of the study methods.
Explore potential weaknesses of the study design (qualitative)			Suggestions for improvements have been identified, for example, Reduce and alter questionnaire material Remove 12-week follow-up assessment as there was most missing data (23%) Remove medication side-effects subscale of ELDQOL Develop and add epilepsy knowledge measure Provide several completion formats for follow-up questionnaires Option to complete an online questionnaire or answer questions over the phone Minimise missing data at 20 weeks 20-week questionnaire will be completed during a study visit with the research nurse Understand how booklet is used by participants and carers Conduct observations of booklet use at 20-week follow-up
Explore the acceptability of the Beyond Words booklet (qualitative)	Feasibility criterion 6: good acceptability of the intervention among participants, carers and health professionals	*Criterion met*	High levels of acceptability of the booklet in intervention group Use of booklet perceived to be feasible in routine care
To explore the feasibility of collecting resource use and quality of life data (quantitative)	Feasibility criterion 7: collection of resource use and quality of life data is feasible, and the increase in treatment cost will be minimal.	*Criterion partially met* amendments: Costs may be lower in a full-scale trial as the training costs are apportioned per participant.	Good rates of data completion and minimal data cleaning was required. The mean per participant cost of the intervention was £122. Preliminary cost-effectiveness analysis indicates no significant difference between the two groups.
Monitor the intervention's patterns of use postrandomisation, and explore whether other resources on epilepsy have been used (quantitative)			86% of participants used the booklet at least once more at home. The number of participants in the intervention group (n=21) reporting to have used the intervention on the follow-up questionnaires decreased over time from 15 (4 weeks) to 12 (12 weeks) to 10 (20 weeks) Minimal use of other education/information resources

ELDQOL, Epilepsy and Learning Disabilities Quality of Life Scale.

Quantitative data analysis was undertaken on Stata V.13 (StataCorp. Stata Statistical Software: Release 13. College Station, Texas: StataCorp LP; 2013). Most analysis was descriptive to evaluate the flow of patients through the study, the proportion of missing questionnaires, demographics of participants, use of the picture booklet and seizure control. For the primary outcome, ELDQOL, the variability of the scores for the four subscales were estimated at each follow-up time point in line with Cocks and Torgerson.^36^ Cohen's d effect sizes for the difference between the two groups were calculated. The 80% CIs of the effect sizes were examined to determine whether they excluded an effect size of zero, that is, an indication of a difference between the two groups, and included the target effect size, that is, a 10% advantage for the intervention group.

For the health economic analysis, levels of resource use associated with the booklet, intervention staff training, face-to-face meetings (including travel), and the 2-week follow-up call were recorded, and unit costs (£GBP for the 2013–2014 financial year) were subsequently assigned to all items.^37–40^ The total costs were then apportioned across all participants in the intervention arm, in order to estimate mean per participant cost of the intervention. In order to estimate overall cost to the NHS, Personal Social Services (PSS), and informal care, the carer was asked to complete a resource use questionnaire at baseline and 20-week follow-up.

Health-related quality of life was estimated using the EuroQol EQ-5D-5L questionnaire,^34^ a generic preference-based measure. EQ-5D-5L data were converted into utility scores (a scale where zero is equal to death and one is full health)^41^ using a mapping approach based on the three-level version.^42^ Quality-adjusted life year (QALY) scores were subsequently calculated using the area under the curve approach.^43^ As this was a feasibility study, the main focus was on completion rates. A preliminary within-trial analysis (using bivariate regression,^44^ based on a complete-case analysis^45^) was also conducted over the 20-week follow-up period, though these results need to be treated with caution due to small numbers. The incremental cost-effectiveness ratio (ICER), defined as mean incremental cost/mean incremental effect, was subsequently estimated.^41^ NICE generally deems interventions which have an ICER below £20 000 per QALY as cost-effective.^46^

The semistructured interviews were transcribed and analysed using thematic content analysis^47^ on NVivo V.11. Two members of the research team coded the interviews, with 30% of the interviews being coded by both researchers. Codes were compared and then discussed, and themes were derived through discussion.

## Results

Four out of seven criteria used to determine the feasibility of a full-scale trial were fully met (table 2). Three criteria were partially met. Amendments to the study design and methodology have been identified and would be implemented in a definitive trial. The participant flow diagram for the study can be seen in figure 1.

### Recruitment

Recruitment began in July 2014 and was originally scheduled to be completed by December 2014. The recruitment period was extended by 4 months to compensate for logistical issues that caused delays at the study onset. The target of 40 participants was met in April 2015, without delaying the planned study completion date. All participants and carers chose to be visited at home by the researcher. Follow-up data collection continued until September 2015, and this is when participants' involvement in the study ended.

The first feasibility criterion was to randomise 60% of eligible patients. The study recruited 25% of all screened patients (n=160) and 34% of screened patients who were eligible (n=118). Despite not meeting this feasibility criterion, the study recruited to target without delay to the study end date.

The most common reasons for being excluded from the trial was the carer reporting that the patient would be unable to use the booklet due to communication issues or an absence of seizures within the past 12 months. There was a range of reasons for people declining to take part, although approximately half of the carers/patients did not give a reason.

The characteristics of participants across the two groups can be seen in table 3. There are some differences between the groups. In particular, the intervention group had had their epilepsy diagnosis for fewer years. They were also slightly younger and received fewer hours of care from their carer.

**Table 3:** Demographics of participants

	Control group (n=19)	Intervention group (n=21)
Gender		
Female:male	10:9	13:8
Age		
Mean (SD)	44.68 (14.53)	39.00 (12.65)
Ethnicity		
White	19	19
Bangladeshi	0	1
Black—other	0	1
Years since diagnosis*		
Mean (SD)	39.31 (16.22)	23.38 (16.49)
Accommodation		
Family home	6	3
Own property	0	1
Registered care home	5	7
Supported accommodation	8	9
Tenancy	0	1
Carer relationship to participant		
Family member: paid carer	6:13	5:16
Number of hours of care per week provided by carer		
Median (IQR)	37 (35–168)	15 (10–168)

*Missing data=14.

### Data collection

The proportion of completed study measures was 85% with most missing data at T2 (23%), thus meeting the second feasibility criterion overall (at least 80% of study measures completed). The proportion of missing data was similar across the two groups. Two participants did not complete any follow-up questionnaires (T1–T3), but did not ask to withdraw from the study (despite the opportunity to do so).

### Discontinuation rates

No participants withdrew from the trial. One participant died over the course of the study, unrelated to study participation. One participant was considered not to have received the intervention as they refused to look at the booklet with the research nurse and with their carer at a later date. An intention-to-treat analysis was carried out, including these participants. Therefore, the third feasibility criterion (discontinuation rates under 20%) was met.

### Intervention effect on primary and secondary outcomes

The intervention group generally reported higher scores at baseline (T0) compared with the control group on the ELDQOL, indicating poorer quality of life. Therefore, adjusted means for the follow-up time points were calculated controlling for baseline differences. Effect sizes were then calculated for the group difference for the outcome measures, along with 80% CIs. Table 4 shows the baseline mean scores and SDs, adjusted T1–T3 mean scores and CIs, along with effect sizes and CIs. Examination of effect sizes and CIs indicated a signal of a positive effect from the intervention on the behaviour and mood subscales of the ELDQOL at T1 and T3. On subscales for behaviour, mood and seizure severity (secondary outcome) at T2, there was indication of improved scores in the control group. This finding is unexpected and there is no theoretical explanation; the most likely reason for this finding is the relatively small sample size at T2 due to the largest proportion of missing data being at this time point (23%).

**Table 4:** ELDQOL mean scores, SDs and 95% CIs with T1–T3 adjusted for baseline

	N	Control group			N	Intervention group			
		Mean	SD	95% CIs		Mean	SD	95% CIs	Effect size of the group difference (80% CI)
ELDQOL seizure severity									
T0 (baseline)	12	27.50	8.29	–	11	29.25	9.37	–	–
T1 (4 weeks)	10	26.42	–	22.46 to 30.39	8	26.66	–	22.50 to 30.82	0.04* (−0.17 to 0.25)
T2 (12 weeks)	11	24.26	–	20.27 to 28.25	9	29.17	–	25.01 to 33.33	0.79* (0.43 to –1.15)
T3 (20 weeks)	13	25.62	–	21.68 to 29.56	8	27.06	–	22.68 to 31.44	0.21* (−0.02 to 0.44)
ELDQOL side effects									
T0 (baseline)	11	71.56	4.93	–	15	65.99	10.75	–	–
T1 (4 weeks)	12	65.66	–	50.02 to 72.29	17	65.98	–	60.84 to 71.12	0.03* (−0.13 to 0.19)
T2 (12 weeks)	13	66.57	–	60.10 to 73.05	15	65.15	–	59.84 to 70.46	0.12† (−0.06 to –0.30)
T3 (20 weeks)	11	64.54	–	57.65 to 71.43	14	64.87	–	59.60 to 70.14	0.03* (−0.14 to –0.20)
ELDQOL behaviour									
T0 (baseline)	19	14.36	3.25	–	21	15.96	5.33	–	–
T1 (4 weeks)	15	15.48	–	13.92 to 17.03	19	14.67	–	13.29 to 16.06	0.26† (0.07 to 0.45)
T2 (12 weeks)	15	14.39	–	12.83 to 15.95	16	15.25	–	13.78 to 16.71	0.28* (0.08 to 0.48)
T3 (20 weeks)	16	15.86	–	14.33 to 17.39	16	14.50	–	13.03 to 15.96	0.43† (0.21 to –0.65)
ELDQOL mood									
T0 (baseline)	19	28.27	6.10	–	20	30.72	7.31	–	–
T1 (4 weeks)	15	31.07	–	28.73 to 33.42	18	29.53	–	27.34 to 31.71	0.32† (0.12 to 0.52)
T2 (12 weeks)	15	30.64	–	28.27 to 33.00	16	31.62	–	29.32 to 33.93	0.21* (0.02 to 0.40)
T3 (20 weeks)	16	31.08	–	28.78 to 33.38	17	30.05	–	27.85 to 32.24	0.22† (0.04 to 0.40)

Higher scores reflect poorer quality of life.

*Effect size in favour of the control group.

†Effect size in favour of the intervention group.

ELDQOL, Epilepsy and Learning Disabilities Quality of Life Scale.

Participants reported difficulties completing the ELDQOL medication subscale. Respondents were often unable to determine whether a behaviour was caused by epilepsy medication (and could therefore be identified as a side effect of epilepsy medication), or was due to other medication or other reasons. Furthermore, the seizure subscale was only completed for participants who had experienced a seizure over the previous 4 weeks. The sample size was often very low: 8–11 people in the intervention group (n=21) and 10–13 people in the control condition (n=19).

The fourth feasibility criterion (likelihood of improvement in quality of life) can therefore be considered to be partially met: indication of an effect of the intervention on the two ELDQOL subscales (behaviour and mood), at two out of three time points (T1 and T3), with the most complete data.

Seizure control was a secondary outcome, and was assessed through completion of a seizure diary during the 20-week study period. If participants had not experienced seizures over the previous 4 weeks, then they did not complete the ELDQOL seizure severity subscale at each time point. A sizeable minority of participants reported having no seizures over the previous 4 weeks: a mean of 38% in the intervention group compared with 25% in the control group. Similarly, more intervention group participants than control group participants experienced no seizures over the course of the study (33% compared with 16%). As would be expected from these results, the intervention group reported fewer seizures over the 20-week study period than did the control group (13.78 (95% CI −0.36 to 35.61), compared with 17.63 (95% CI 1.73 to 25.83)).

### Acceptability and feasibility of methodology and intervention

Interviews with 15 sets of participants and/or carers took place at the 20-week time point and occurred between January and September 2015. Eleven interviews took place with both participant and carer(s), one participant and their carer were interviewed separately, two interviews took place with the participant only and one interview took place with the carer only. Interviews with four health professionals took place between February and November 2015. The health professionals were all involved in the study, although this varied from close to minimal involvement.

Table 5 shows the themes and subthemes regarding the study methods and intervention from the interviews with participants and carers, and the results indicate that feasibility criteria 5 and 6 were met. Interviewees were generally very positive about their experience of the WIELD project and would recommend participating in the study to others. Some areas for improvement were suggested, for example, offering online questionnaire completion, and reducing the length of the questionnaires. Participants and carers also identified specific questions or subscales, which they found difficult to complete, and which tended to coincide with missing data.

**Table 5:** Key themes about acceptability and feasibility from the interviews with participants and carers

Themes	Subthemes
Feasibility and acceptability of participation	Most people enjoyed taking part in WIELD and would recommend it to others. Most people understood the need for random allocation and accepted this was part of the study. Study information explained the study well and was suitable for most participants. Study visit went well and everything was explained clearly.
Feasibility and acceptability of data collection methods	Completing the questionnaires four times was fine for most people. Most participants were involved in completing the questionnaires. Most people felt that the questions and language were clear but there was some room for improvement. Postal questionnaires were fine for most people but it would be helpful to have options to complete the questionnaire online, on the phone or at a visit. Some carers and participants sometimes found it challenging to find the time to complete the questionnaires. Some people completed the seizure diary but others did not. Some people thought the questionnaires were quite long but this was not a problem for most people. Specific questions could be difficult to answer.
Use and acceptability of the booklet	A minority of carers and participants were already familiar with Beyond Words. Most participants engaged with the booklet and could identify benefits. Control participants had differing views on the booklet after first looking at it. The booklet was used in different ways.
Feasibility of routine use of the booklet	Mixed feelings about level of support needed to use the booklet. Need to be aware if person with learning disabilities is anxious as the booklet could make this worse. The booklet could be implemented in the health and social care system. The booklet could be useful for other people with epilepsy. The booklet is useful for people with difficulties with verbal communication. The booklet may be most useful when someone has just been diagnosed. The booklet may be particularly helpful to explain epilepsy to others. The time needed to use the booklet could be a barrier.

WIELD, Wordless Intervention for Epilepsy in Learning Disabilities.

Participants and carers reported that they had received little information about epilepsy in the past, which was accessible for people with learning disabilities. The booklet was generally received very positively by participants and carers, and participants reported relating the booklet to their own experiences. Participants and carers felt that the epilepsy-related conversations prompted by the booklet were helpful, and revealed new information. Several participants also noted that the booklet helped them to further understand epilepsy and to explain their condition to others. Participants with well-controlled epilepsy felt that the intervention may be more appropriate for people who had recently been diagnosed, or had difficulties understanding or managing their epilepsy. Some carers and participants felt that they needed the guidance provided in the intervention session in order to be able to use the booklet, whereas others felt that they would be able to use the booklet without this.

The interviews with health professionals largely supported the observations made by participants and carers and suggested ways to improve the set-up and recruitment phase of the study. Participating health professionals felt that carers needed support to use the booklet appropriately. There were mixed views about the group of participants who this intervention may be most effective for. The booklet was reported to be more difficult to use with non-verbal participants, although the health professionals acknowledged that the booklet was designed for use with this population and this group often had the most severe epilepsy. It was also suggested that although the booklet had the potential to be useful for all people with learning disabilities and epilepsy, it may be most useful for people who had recently been diagnosed.

In addition to the qualitative data, quantitative data about the use of the booklet during the study were collected. The intervention session took place in one sitting and took ∼20 min. The majority of people in the intervention group used the booklet at least twice after the intervention session (n=14), with three participants never using the booklet at home. The number of people reporting to have used the booklet at home since the previous questionnaire decreased over the course of the study from 15 at T1, to 12 at T2, and to 10 at T3. The frequency and time spent using the booklet also decreased over time. At T3 those who were still using the booklet reported to be doing so with a wider range of people than at T1. There were no reported harms or unintended effects in either group.

### Health economic analysis

Across the 21 intervention patients, the mean per participant cost of the intervention was estimated to be £121.56, where this was composed of training costs (£28.42 per participant), an initial meeting cost (£72.14 per participant, based on a 21 min meeting with travel time and costs included), a follow-up phone call (£11.00 per participant) and the cost of the book (£10.00). The seventh feasibility criterion states that a minimal increase in treatment cost should be evident; therefore, we judge this to be partially met. However, we estimate that given the research context, this cost is significantly higher than what it would cost to use the booklet in routine care (eg, to be conservative all training costs were apportioned across the small number of participants in the intervention arm of the study). Paired cost and EQ-5D-5L data were available for 29/40 participants at the 20-week follow-up point (16 intervention, 13 control), demonstrating that it was generally feasible to collect the economic data in the devised manner. All subsequently reported results are based on these 29 participants. Over the 20-week follow-up period, mean NHS costs (excluding the cost of the intervention) were £616.79 and £698.46 for the intervention and control arm, respectively. Based on the regression analyses, which adjusted for baseline costs and included the cost of the intervention, over the 20-week follow-up period, mean NHS costs were estimated to be £87.08 (95% CI −375.77 to 549.94) higher for the intervention group compared with the control group.

Total mean NHS, PSS and informal care costs (which included residential costs, where applicable) were substantially higher and estimated to amount to £36 656.96 for the intervention and £45 430.40 for the control arm, respectively. The mean cost difference between groups was not estimated to be significant (−£2663.39, 95% CI −8480.29 to 3151.51). Quality of life as assessed by the standard EQ-5D-5L questionnaire can be seen in table 6. The mean QALY difference was −0.006 (95% CI −0.050 to 0.038). The corresponding EQ-5D visual analogue scale (0–100 with higher scale indicating better quality of life) was also similar between treatment groups. It can therefore be seen that there was no significant difference between either the mean costs or QALY score for the intervention group compared with the control group.

**Table 6:** EQ-5D-5L mean scores, SDs and 95% CIs

	Control group				Intervention group			
	N	Mean	SD	95% CIs	N	Mean	SD	95% CIs
EQ-5D-5L index								
T0 (baseline)	19	0.461	–	0.301 to 0.621	21	0.611	–	0.480 to 0.743
T1 (4 weeks)	15	0.494	–	0.291 to 0.696	18	0.643	–	0.459 to 0.692
T2 (12 weeks)	14	0.497	–	0.263 to 0.731	16	0.594	–	0.436 to 0.752
T3 (20 weeks)	13	0.622	–	0.404 to 0.840	17	0.661	–	0.504 to 0.818
QALY	13	0.219	–	0.139 to 0.299	17	0.241	–	0.188 to 0.294
EQ-VAS								
T0 (baseline)	19	77.37	16.45	–	21	70.62	27.30	–
T1 (4 weeks)	15	71.33	21.25	–	18	76.89	23.84	–
T2 (12 weeks)	15	83.67	17.78	–	16	74.88	20.17	–
T3 (20 weeks)	15	78.20	21.53	–	16	76.69	25.52	–

QALY, quality-adjusted life year; VAS, visual analogue scale.

## Discussion

### Principal findings

RCTs are the gold standard for evaluating interventions and services. This study is the first completed RCT addressing the outstanding information and self-management needs of people with learning disabilities and epilepsy. Recruitment was extended by 4 months and achieved the target, without delaying the study's end date. Eighty-five per cent of study measures were completed and attrition was minimal (one death unrelated to study involvement and no other drop-out). A potential effect of the intervention on two ELDQOL subscales was found at 4 and 20 weeks. The acceptability and feasibility of the methodology and intervention were high among participants, carers and health professionals. Collection of economic data was found to be feasible. All feasibility criteria were fully met (n=4) or partially met (n=3) and qualitative and quantitative findings indicated improvements to the recruitment and data collection procedures, thus confirming the feasibility of undertaking a definitive trial.

### Strengths and weaknesses

The strengths of this study were the rigorous randomised controlled evaluation of the feasibility of this pictorial intervention. This approach has very rarely been adopted before in the context of learning disabilities research.^15^ Second, there were high levels of public and patient involvement, including people with learning disabilities and their carers, which significantly strengthened the study design, management and dissemination of the study findings.^48^ ^49^ It is hoped that the successful delivery of this study paves the way for further research in this area with similarly robust methodology, supported by continuous stakeholder involvement.

However, the following limitations need to be considered. First, the proportion of people with an epilepsy diagnosis increases with the severity of learning disabilities, with a recent prevalence estimate of 42% in people with ‘more severe’ learning disabilities.^5^ Only four study participants had limited or no verbal communication (two in each group), which is not representative of this group of patients. During recruitment, it became clear that many carers of patients with severe learning disabilities and/or non-verbal communication felt that they did not meet the inclusion criteria of being able to interact with or follow the booklet's story, although clinicians had initially identified the patient as eligible during screening (n=21). As the booklet was designed to be used by people with a wide range of learning disabilities and communication abilities, this would need to be addressed in a full-scale trial by ensuring that the initial material presented to carers highlights the study's and intervention's suitability for people with severe learning disabilities and/or limited verbal communication. Second, this study relied on proxy completion of the questionnaires, in order to standardise questionnaires across all participants. From the qualitative data, it is evident that carers involved people with learning disabilities to different degrees and that some participants with mild learning disabilities completed the questionnaires independently. There is evidence to suggest that there are differences in the responses of patients, family carers and paid carers on questionnaires of epilepsy-related concerns.^50^ ^51^ In future work, the variation across different respondents could be examined.

### Research in context

People with learning disabilities are under-represented in clinical trials, which results in a limited knowledge base about effective healthcare interventions in a population already experiencing health disparities.^12^ ^19^ There are modifications and adaptations that can be made to study designs and procedures to minimise obstacles including accessible information materials (eg, easy-read formats were successfully used in WIELD—see online supplementary material), simplified consent procedures, modified procedures to allow support from carers and simplified data collection materials.^19^ People with learning disabilities are positive about participating in research and highlight the importance of researchers understanding their experiences and opinions.^52^

10.1136/bmjopen-2016-012993.supp2 Supplementary WIELD

Two key factors in this study facilitated successful recruitment and high levels of acceptability of the design. First, the study procedures and materials were carefully designed with the feedback of people with learning disabilities, carers, members of the public and health professionals, which resulted in improved accessibility. Second, the design of this study was closely aligned with local epilepsy and learning disability services, which facilitated recruitment. It is important for clinical trials to be embedded within the relevant health services to ensure successful recruitment and likelihood of potential implementation.^53^ ^55^ Services across England for people with learning disabilities and epilepsy vary greatly;^15^ the clinical care pathway in different NHS Trusts may affect recruitment in a full-scale trial and routes to implementation in routine care. Therefore, future research and dissemination would need to continue to work collaboratively with local services, and explore different ways of using the booklet and how this may, or may not, align with local practice.

A recent scoping review of self-management interventions for people with learning disabilities and epilepsy^25^ identified a nurse-led intervention that is currently underway,^55^ and three completed small-scale studies.^56–58^ The results of the completed studies are promising and suggest that a self-management intervention could be appropriate and beneficial for people with learning disabilities and epilepsy. However they are underpowered, did not use a RCT design and did not assess outcomes on quality of life or seizure control.

### Implications for clinicians and policymakers

Very few of the study participants had ever received information about epilepsy that was adapted to their communication ability. Much of the information provided by health professionals was not easily accessible, and was aimed at carers. The booklet was received positively and various benefits were noted. This suggests that there is a need for tailored education and self-management information for people with learning disabilities and epilepsy, which is currently unmet. The feasibility findings are promising, but the results of a definitive trial are necessary to demonstrate whether the proposed intervention meets these needs, and improves outcomes.

The qualitative data suggested that some participants experienced improvements from the intervention in their epilepsy-related knowledge, confidence and anxiety. There is currently no measure that assesses these constructs in people with learning disabilities and epilepsy. Further research could explore this to understand more about the needs of this population, and how to meet these needs.

This study did not explore the fidelity of the intervention; in order to fully evaluate this intervention, it would be crucial to ascertain whether the intervention is used by participants and carers as recommended, and to explore and understand the underlying mechanisms of booklet use. A process evaluation approach using ethnographic methods would be informative to observe how the booklet is used, and what the ‘critical ingredients’ of this intervention may be.

## Conclusions

The WIELD study was completed with successful recruitment and minimal missing data. Four out of seven feasibility criteria were fully met, and three criteria were partially met, thus confirming the feasibility of undertaking a definitive trial. Acceptability of the study methods and intervention was high among participants, carers and health professionals. By developing and testing methods to reduce the number of follow-ups, extend the recruitment period, develop study information material to increase the involvement of participants with severe learning disabilities and assess epilepsy knowledge, recruitment rates could increase and the effects of the intervention could be measured more accurately. Given the success of the feasibility study and robust evidence to address any potential issues, it is clear that a definitive trial is both feasible and necessary to address important gaps in evidence-based practice for people with learning disabilities and epilepsy.
