# Supplementary material for: Wordless intervention for people with epilepsy and learning disabilities (WIELD): a randomised controlled feasibility trial
Source: BMJ Open. 2016 Nov 10;6(11):e012993. doi: 10.1136/bmjopen-2016-012993 (PMC5128894; doi:10.1136/bmjopen-2016-012993)
Supplement: Supplementary easy-read information sheet [file bmjopen-2016-012993supp_easy-read-info-sheet.pdf]

Information about a study in this epilepsy clinic

Study title: WIELD (wordless intervention for epilepsy in learning disabilities)

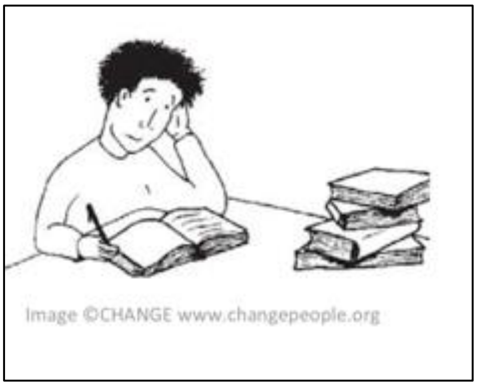

- Hello, we are researchers and doctors doing a study.
- A researcher is a person who finds out new things.
- A study is a way of finding things out.

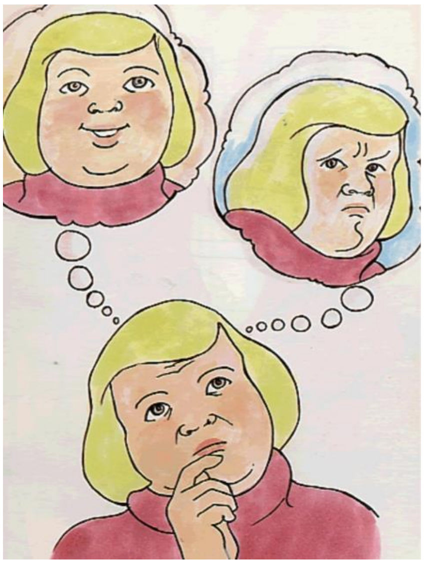

© Sheila Hollins
www.bookbeyondwords.co.uk

- **Would you like to be in the study?**

- This leaflet tells you about the study.

It will help you decide if you want to be in the study.

UNIVERSITY OF WEST LONDON Connected. University of Hertfordshire UH UNIVERSITY OF CAMBRIDGE UEA University of East Anglia

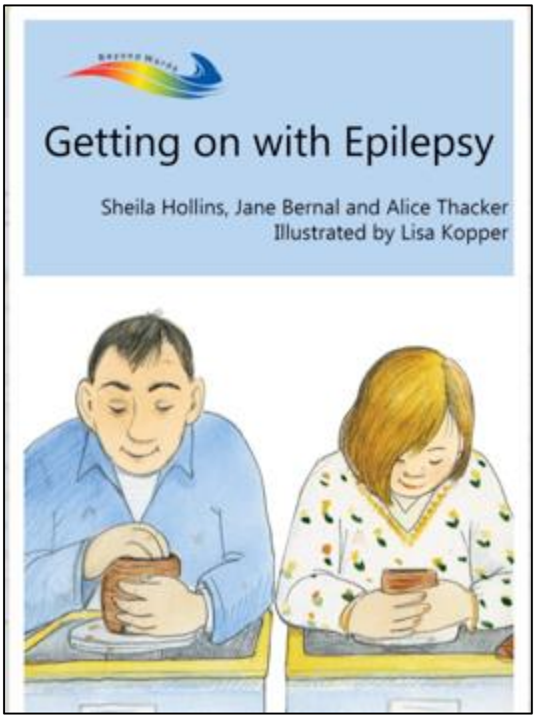

What is the study about?

- The study is about epilepsy.
- We want to find out if a book with pictures can help people with learning disabilities to understand and manage their epilepsy.
- We are doing a small study with a small number of people. It will help us decide if a larger study is possible.

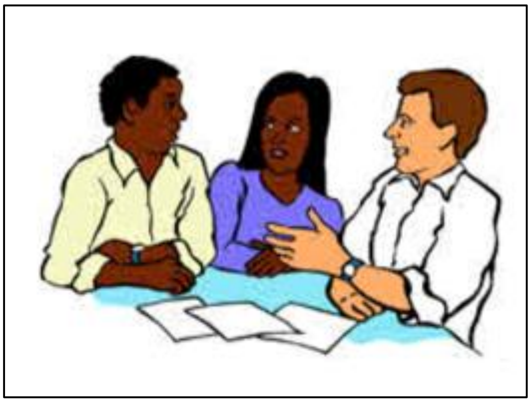

- The larger study will tell us if the book is helpful for people with learning disabilities who have epilepsy.

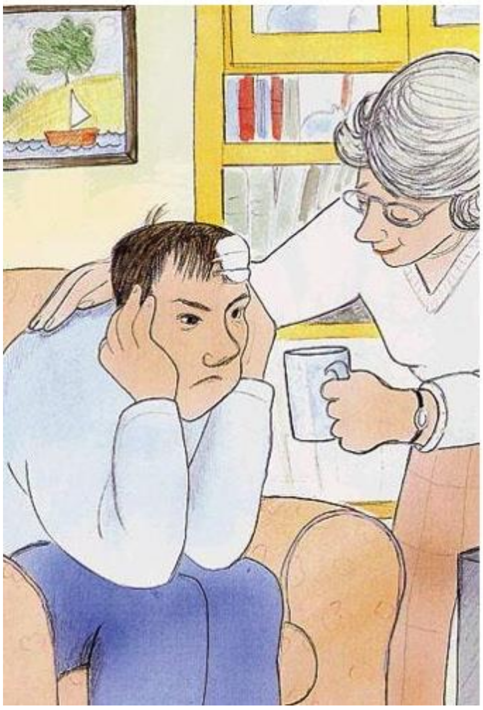

© Sheila Hollins
www.bookbeyondwords.co.uk

Why are we doing this study?

- We know that people with learning disabilities can find it hard to understand and manage their epilepsy.

- We want to know if a book with pictures is helpful.

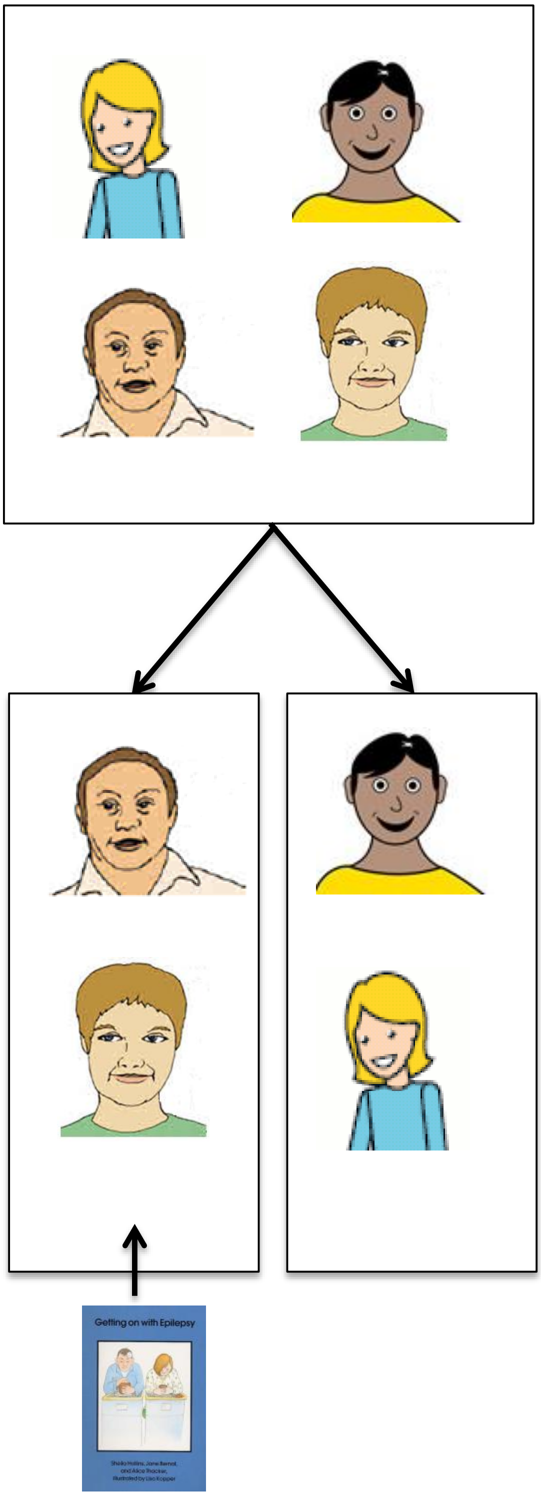

What happens in the study?

- We want to know about you and your epilepsy.
- We will put people into two groups by chance (randomly).
- In one group, people will be given a book, to use with the nurse and at home.
- In the other group, people will not be given a book to start with. They can use the book later.
- We want to know what you think about using the book (if we give you one).

UNIVERSITY OF WEST LONDON Connected. University of Hertfordshire UH UNIVERSITY OF CAMBRIDGE UEA University of East Anglia

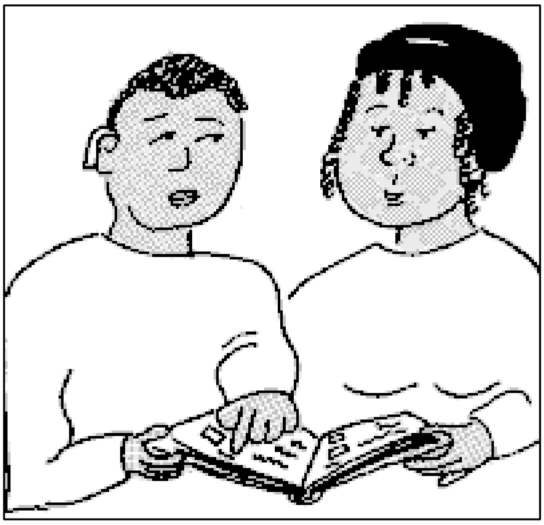

- We will also give you a questionnaire. We want you to answer the same questionnaire 4 times.

- You can do this with your carer or family.

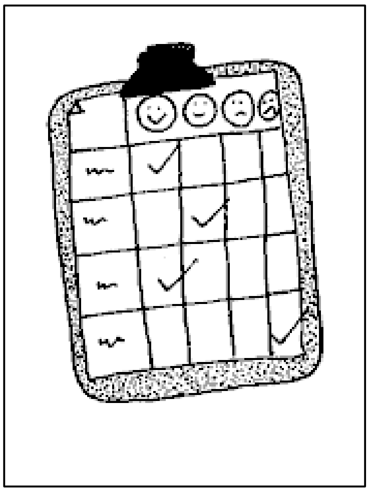

- We may also ask you some questions in an interview.

- We will show you some pictures to help you think about the questions.

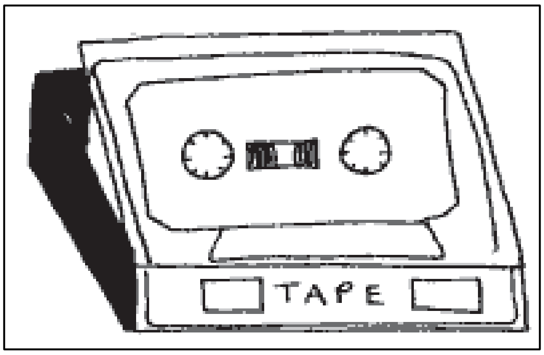

- We may record what you say on a tape.

- If you don't mind, we will use a tape recorder. It will help us remember what you have said.

What will we do after the study?

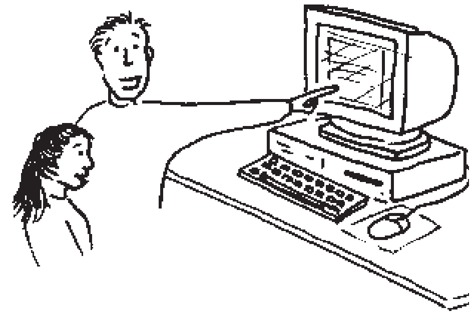
Image © CHANGE www.changepeople.org

- Afterwards, what you have told us will be put in a computer.
- We will not write down your names. We will use pretend names instead.

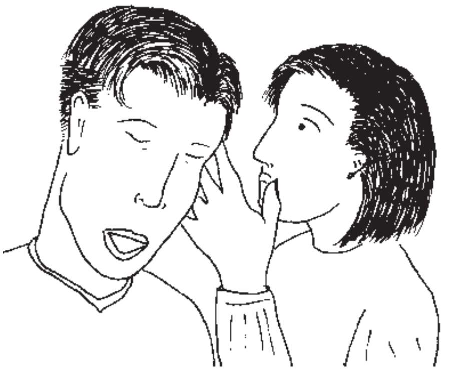
Image © CHANGE www.changepeople.org

- Everything you say to us is private.
- We will tell others what we found out. But we won't tell them who said it.

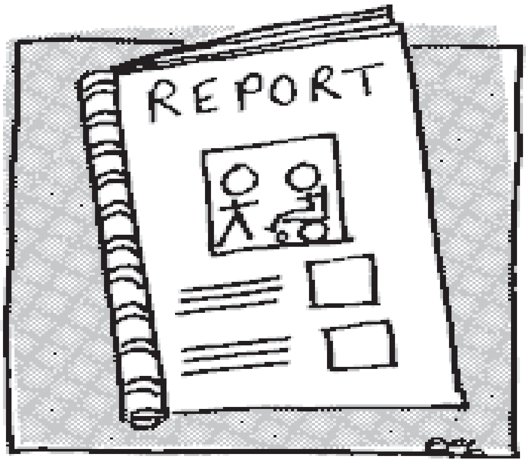

- We will also write in magazines about what we have found out.

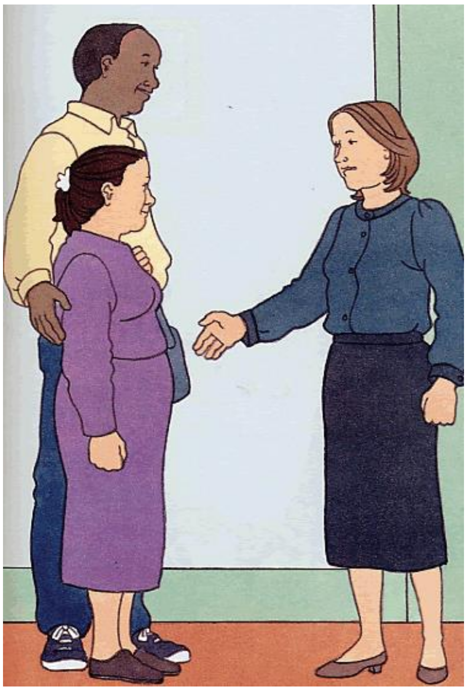

© Sheila Hollins
www.bookbeyondwords.co.uk

Can someone come with me?

- Yes, your family or carer can come to the epilepsy clinic with you.

Do I have to be in the study?

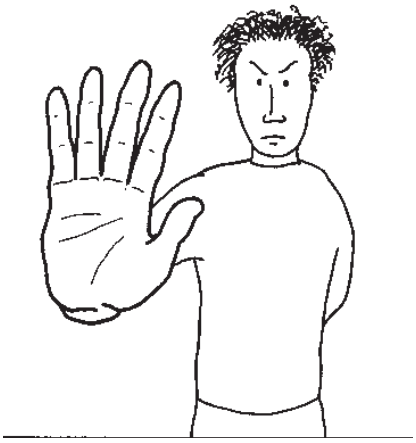

Image © CHANGE www.changepeople.org

- Being in the study is up to you.
- It is your choice.
- If you do not want to do it, that's ok.

- You can stop doing the study if you do not like it. You can stop at any time.

- Nobody will be annoyed if you say no or if you stop.

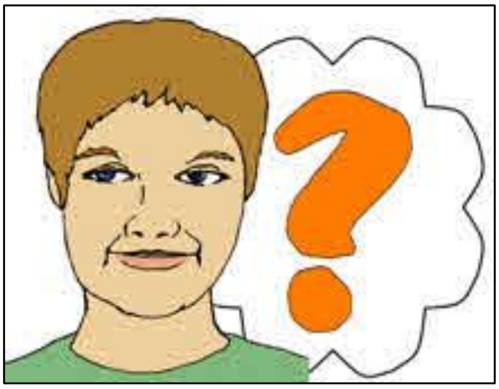

What will happen now?

- You can call or write to us to ask questions about the study.

- If you want to know more about the study, you or your carer or family can call Silvana. Silvana is a researcher.

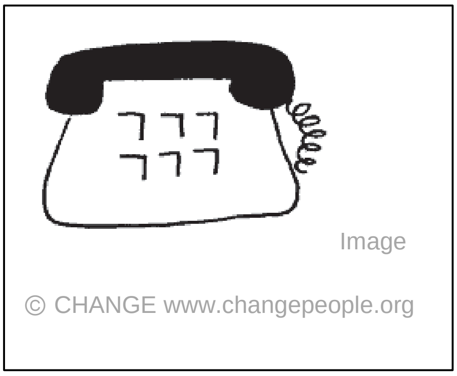

- Her phone number is:
  01707 284494

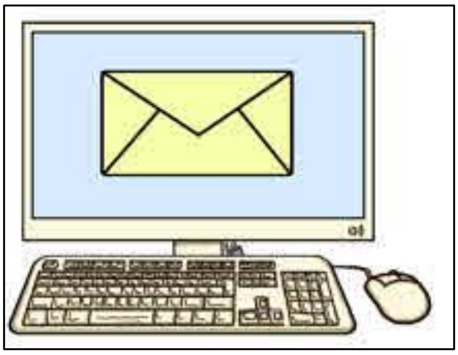

- Or you can send Silvana an email:
  s.mengoni@herts.ac.uk

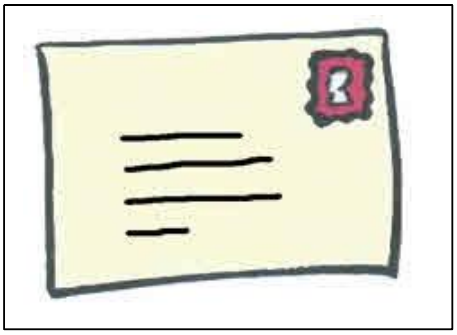

- Or you can write to Silvana.

Her address is:
Silvana Mengoni
CLiCIR
School of Life and Medical Sciences
University of Hertfordshire
College Lane Campus
Hatfield AL10 9AB
