# Supplementary material for: Wordless intervention for people with epilepsy and learning disabilities (WIELD): a randomised controlled feasibility trial
Source: BMJ Open. 2016 Nov 10;6(11):e012993. doi: 10.1136/bmjopen-2016-012993 (PMC5128894; doi:10.1136/bmjopen-2016-012993)
Supplement: Supplementary WIELD [file bmjopen-2016-012993supp_WIELD.pdf]

A randomised controlled feasibility trial of the Books Beyond Words intervention to improve the management of epilepsy in people with learning disabilities

Trial Protocol

Clinical trial registration number: ISRCTN80067039

REC reference number: 14-WA-0135

Version number: 1.0

Funder: National Institute for Health Research, Research for Patient Benefit

Sponsor: Hertfordshire Partnership University NHS Foundation Trust

Table of contents

1. General Information 5

2. Summary 7

2.1. Lay Summary 7

2.2. Scientific Summary 8

2.3. Design Overview 8

2.4. The Research Team 9

3. Introduction 11

3.1. Background and rationale 11

3.2. Aims and objectives 14

4. Methods 16

4.1. Design 16

4.2. Setting 16

4.3. Participants 16

4.4. Study flow-chart 17

4.5. Intervention 18

4.5.1. How the Books Beyond Words intervention will be used 18

4.5.2. Training 19

4.6. Control Group 19

4.7. Outcomes 19

4.7.1. Quantitative assessment 20

4.7.2. Qualitative assessment 21

4.8. Procedure 22

4.8.1. Trial Setup 22

4.8.2. Screening 22

4.8.3. Consent and baseline assessment 23

4.8.4. Randomisation 24

4.8.5. Intervention condition 24

4.8.6. Control condition 25

4.8.7. Qualitative interviews 25

4.8.8. Study termination 25

4.9. Proposed sample size 25

4.9.1. Feasibility of recruitment 26

4.10. Statistical analysis 26

4.10.1. Quantitative assessment 26

4.10.2. Qualitative assessment 27

4.11. Health economic analysis 27

4.12. Data management 27

4.13. Write-up and dissemination 28

5. Timeline 29

6. Ethical considerations 30

6.1. Consent 30

6.2. Data protection and confidentiality 30

6.3. Other ethical considerations 31

7. Expected output and impact of research 32

8. Sponsorship and indemnity 33

8.1. Sponsor's details 33

8.2. Indemnity arrangements 33

9. Study Management and finance 34

9.1. Study management 34

9.2. Trial Steering Committee 34

9.3. Reference group 34

9.4. Financial responsibility and management 35

10. References 36

11. Abbreviations 39

12. Appendices 40

12.1. Collaboration agreement 40

12.2. RfPB funding confirmation 40

12.3. RfPB reviewer comments 40

12.4. Books Beyond Words 41

12.5. Case Report Form ... 42
12.6. Questionnaire baseline ... 42
12.7. Questionnaire 4 week follow-up Intervention Group ... 42
12.8. Questionnaire 4 week follow-up Control Group ... 42
12.9. Questionnaire 12 week follow-up Intervention Group ... 43
12.10. Questionnaire 12 week follow-up Control Group ... 43
12.11. Questionnaire 20 week follow-up Intervention Group ... 43
12.12. Questionnaire 20 week follow-up Control Group ... 43
12.13. Health Professional Information Sheet ... 44
12.14. Health Professional Consent Form ... 44
12.15. Interview Schedules ... 44
12.16. Interview Schedule – easy read version ... 44
12.17. Seizure diary ... 45
12.18. Carer Invitation Letter ... 45
12.19. Carer/Consultee Information Sheet ... 45
12.20. Participant Invitation Letter ... 45
12.21. Participant Information Sheet ... 46
12.22. Participant Consent Form ... 46
12.23. Carer/Consultee Declaration Form ... 46
12.24. GP Information Letter ... 46
12.25. Timetable ... 47

1. General Information

This document provides details regarding the setting up of, conduct, and analysis of the National Institute for Health Research (NIHR), Research for Patient Benefit (RfPB) funded study, “A randomised controlled feasibility trial of the Books Beyond Words intervention to improve the management of epilepsy in people with learning disabilities”. Every care was taken in its drafting, by corrections or amendments. Approval by NHS ethics and R&D is necessary. All investigators involved in the study should be familiar with the most up to date version of the protocol.

Compliance

This study will be conducted in compliance with the Data Protection Act 1998[1], the Mental Capacity Act 2005[2] and the principals of the Declaration of Helsinki (1964) as revised in Tokyo (2004)[3]. All investigators involved in the study will be expected to have completed Good Clinical Practice (GCP) training.

Sponsor

Hertfordshire Partnership University NHS Foundation Trust (HPUT) will sponsor this study. The responsibilities of the Sponsor are described in appendix 1. The University of Hertfordshire (UH) will be a partner in the conduct of this study. As such, collaboration agreements will be exchanged and signed between the trust and the university, specifying responsibilities and financial arrangements (see appendix 1).

Sponsor’s Address: Hertfordshire Partnership University NHS Foundation Trust, 99 Waverley Rd, St. Albans, Hertfordshire AL3 5TL. Tel : 01727 804700.

Contacts:

- Chief Investigator, Professor Bob Gates (Tel: 0208 2312209; Email: bob.gates@uwl.ac.uk)
- Trial Coordinator, Doctor Marie-Anne Durand (Tel: 01707 281157; Email: m.durand@herts.ac.uk)

Funder

This study is funded by the National Institute for Health Research, through the Research for Patient Benefit programme (RfPB PB-PG-0213-30042) (see appendix 2-3).

Key Study Personnel

Chief Investigator: Professor Bob Gates

Co-applicant and study coordinator: Dr Marie-Anne Durand

Research Fellow: to be recruited

Co-applicant: Dr Asif Zia

Co-applicant: Dr David Wellsted

Co-applicant: Dr Garry Barton

Co-applicant: Dr Georgina Parkes

Co-applicant: Dr Howard Ring

Co-applicant: Dr Karin Friedli

Signatures

The Chief Investigator (CI), HPUT and UH have discussed this protocol. The investigators agree to perform the investigations and to abide by this protocol except in case of medical emergency or where departures from it are mutually agreed in writing.

Name: …………………………………   Role: UoH Representative

Signature: ……………….……………   Date: …………………….

Name: …………………………………   Role: HPUT Representative

Signature: ……………….……………   Date: …………………….

Name: …………………………………   Role: Chief investigator

Signature: ……………….……………   Date: …………………….

2. Summary

2.1. Lay Summary

Epilepsy is the most common neurological problem affecting people with learning disabilities. It is more complex, more severe, and leads to more deaths in people with learning disabilities than in the general population who live with epilepsy. People with epilepsy and learning disabilities find it difficult to manage their illness[4]. They do not have easy access to services and struggle to manage repeated seizures. They are often resistant to treatment, which can lead to premature deaths and increased costs[5-7]. The guidelines of the National Institute for Health and Care Excellence recommend that patients with learning disabilities and epilepsy are offered the same standard of care, services and investigations as the general population.

The Books Beyond Words booklet for epilepsy uses images to help people with learning disabilities better understand and manage their condition and improve their quality of life. This intervention has never been formally evaluated and its effectiveness remains unknown. Given the lack of research in this area, it is recommended that this intervention is evaluated in a feasibility trial. This will determine whether a full-scale trial can be undertaken and what sample size, design and methods are most appropriate. The acceptability, potential effectiveness and cost effectiveness of using the booklet for epilepsy will also be explored. Eligible patients with epilepsy and learning disabilities will be randomised to receive either the Books Beyond Words booklet for epilepsy or routine information and care. In the intervention group, the booklet will be used at the Epilepsy Clinic with a Research Nurse and Carer, and later at home with the carer or family. Outcomes will be measured at 1, 3 and 5 months. Semi-structured interviews will also be used to assess feasibility and acceptability.

2.2. Scientific Summary

People with learning disabilities suffer from health inequalities that are, to some extent, avoidable, and are often unrecognised. They have more health problems, and are at higher risk of premature death than the general population[8]. Epilepsy is the most common neurological disorder affecting people with learning disabilities, and is 20 times more common than in the general population[9,10]. The high incidence (16 to 26%), high seizure frequency, resistance to treatments, and associated skills deficit and co-morbidities make the management of epilepsy particularly challenging for people with learning disabilities[11-13]. It is therefore associated with more premature deaths and higher costs in patients with learning disabilities than in the general population of people living with epilepsy[5-7]. The National Institute for Health and Care Excellence and the Royal College of General Practitioners advocate for the recognition of the needs of people with learning disabilities and epilepsy, mostly unmet, so they can be informed and empowered to manage their condition adequately.

Interventions that target the needs of patients with learning disabilities, their carers, and health providers could improve communication, access to services, knowledge and quality of life, therefore contributing to reduce health disparities[4,14]. The Books Beyond Words booklet for epilepsy uses images that tell the story of a young man with learning disabilities and epilepsy, in order to improve understanding, illness management and quality of life of people with learning disabilities. All images have been tested with people with mild to severe learning disabilities who cannot read, to make sure they understand them. The Books Beyond Words booklet was developed jointly by the Royal College of Psychiatrists, with the involvement of people with learning disabilities, their carers, consultant psychiatrists and other medical professionals[15]. To date, this booklet has never been evaluated in controlled contexts or routinely adopted in the NHS.

2.3. Design Overview

Our aim is to undertake a randomised controlled feasibility trial of the Books Beyond Words booklet for epilepsy with qualitative research, testing the feasibility of a large-scale randomised controlled trial in patients with learning disabilities.

The following research questions will guide the study design, methods of data collection and analysis:

- What is the feasibility of undertaking a full-scale randomised controlled trial of the Books Beyond Words intervention for epilepsy in people with learning disabilities?
- Is this intervention acceptable to patients with learning disabilities, carers and health professionals?
- What is the potential effectiveness and cost effectiveness of this intervention?

Patients with learning disabilities who meet the study's inclusion criteria (see section 4 Methods) will be recruited from five Epilepsy Clinics and randomised to either the intervention or control arm of the project. Consent will be obtained with the carer's involvement, who will assess participants' capacity to take part, as specified in the Mental Capacity Act[2]. A Research Nurse will manage the information, consent and baseline assessment. Outcomes will be assessed at 4 weeks, 12 weeks, and 20 weeks post-randomisation.

The use and acceptability of the intervention will be assessed using semi-structured interviews conducted with patients, carers and health professionals (see 4.2 study flow-chart). Although this feasibility study is not powered to assess the effectiveness and cost effectiveness of the intervention, we will undertake a preliminary analysis of both the potential effectiveness and cost-effectiveness of the intervention, which will provide necessary information to design and power a full-scale trial. The information gained from this feasibility trial will then be used to solicit funding for a full-scale trial of the Books Beyond Words booklet for epilepsy.

2.4. The Research Team

The research team is multi-disciplinary and includes clinicians and researchers in Learning Disabilities, Epilepsy, Psychiatry, Psychology, Health Economics, Medical Statistics and Health Services Research. The Chief Investigator, Professor Bob Gates is Emeritus Professor of Learning Disabilities at the University of Hertfordshire; he is also Professor of Learning Disabilities at the Hertfordshire Partnership NHS Foundation Trust, and works part time as Professor of Learning Disabilities, at the University of West London, Institute for Practice, Interdisciplinary Research and Enterprise (INSPIRE). He has been awarded a number of research grants for both quantitative and qualitative studies as well as consultancy work.

Dr Durand is a Health Psychologist and Senior Research Fellow in Health Services Research at the University of Hertfordshire's (UH) Centre for Lifespan and Chronic Illness Research

(CLiCIR). She has solid experience of programme and research management in the NHS, including the evaluation of complex interventions.

Dr Zia is a Consultant Psychiatrist in Intellectual Disability. He is Clinical Director for the Learning Disabilities and Forensic Business Unit, and has managerial responsibility for the consultants and medical staff working in learning disability and forensic services. In relation to quality of services, he works with the University of Hertfordshire to increase research activity within the Business Unit.

Dr Parkes is a Consultant Psychiatrist in Learning Disability and joint Medical Lead for Hertfordshire Partnership NHS Foundation Trust. She is Chair of the Institute of Psychotherapy and Disability, and co-opted member of the Association for psychoanalytic psychotherapy council. She has been an NHS doctor since 1994 and a Consultant since 2007.

Dr Friedli is a Senior Research Fellow in Health Services Research and manages the Clinical Trial Support Network at CLiCIR. She has expert knowledge in the design and management of clinical trials, including governance, trial design, data and trial management.

Dr Ring is a clinical academic Neuropsychiatrist at the University of Cambridge with extensive clinical and research experience in epilepsy and its management in adults with learning disabilities, undertaking a range of research studies funded by grants from NIHR and Epilepsy Action.

Dr Wellsted is a Senior Lecturer in Health Research and Head of UH's CLiCIR. He provides statistical analysis for a range of externally-funded studies. He is a co-applicant on 5 NIHR-funded projects and numerous charity-funded projects.

Dr Barton is a Reader in Health Economics. He has conducted economic evaluations within numerous randomised trials and is a co-applicant on grants totalling more than £10 million.

3. Introduction

3.1. Background and rationale

People with learning disabilities experience a disproportionate burden of ill health and are affected by twice the number of health issues prevailing in the general population: higher incidence of long-term conditions and health risks, poorer health outcomes, and an increased risk of premature deaths[7,16-18]. Epilepsy is one of many long-term conditions affecting people with learning disabilities that burdens their lives and hinders their quality of life[19]. It is considered the most common neurological disorder in people with learning disabilities, with a reported prevalence of 16 to 44%[9,10,17,19,20]. In the general population, the estimated prevalence of epilepsy is between 0.4% and 1%[21].

The clinical management of epilepsy in people with learning disabilities is complex, due to both the clinical characteristics of the condition and physical and cognitive impairments affecting them. Seizures are unpredictable, potentially life threatening, and atypical[22-24]. Further, epilepsy is often severe[13]. Seizures are more frequent than in the general population of people with epilepsy and approximately 70% are refractory to treatments [22,24]. They are less well controlled, and may be accompanied by co-morbid health, mental health, sensory-motor and communication issues[12,25,26]. Poorly controlled epilepsy can severely affect social relationships, work, daily activities, quality of life and mortality[4,27,28]. As a result, young people and adults with learning disabilities and epilepsy face higher risks of premature and avoidable death related to epilepsy, than the general population[29]. In addition, research shows that poorly controlled seizures and seizure frequency are associated with increased costs[30]. Pennington et al. recently demonstrated increased costs in people with epilepsy and learning disabilities[6], with a total estimated cost per patient, per annum, of £64,000 (including accommodation and basic care).

Managing long-term conditions is challenging for all patients, irrespective of their abilities and co-morbidities. However, it becomes even more burdensome and life-threatening for those with learning disabilities, who may not have the knowledge, skills and support required to assess potential risks and cope with recurring seizures, prescribed medications and regular doctor appointments. Research suggests that access to specialist services is poor for people with epilepsy and learning disabilities[8]. For carers and families who are not trained in supporting

self-management, caring for patients with 'dual disabilities', can be extremely challenging and increase the caregiver burden[19]. Despite its high incidence, little is known about the management of epilepsy in people with learning disabilities, and those who support them. Clark et al. examined epilepsy-related knowledge in people with learning disabilities, using an educational package designed to improve understanding. The findings suggest that better knowledge of the characteristics of the disease was associated with improved management and coping, and that the intervention was found useful in adults with mild learning disabilities independently of demographics[14]. Further, research suggests that people with learning disabilities and epilepsy lack skills training adapted to their needs and intellectual abilities and often fail to adequately manage and control their epilepsy[4].

NICE guidelines state that patients with epilepsy and learning disabilities;
'should be empowered to manage their condition as well as possible, and should receive appropriate information and education about all aspects of epilepsy [...] accessible to people with additional needs such as physical, sensory or learning disabilities'[31].

In addition, the guidelines stipulate that adults with learning disabilities, and their carers, should actively participate in developing a personalised care plan and be actively involved in its management[31,32]. According to The Royal College of General Practitioners and Learning Disabilities Observatory, people with learning disabilities should be offered the same standard of care, specialist epilepsy services and investigations as the general population[8]. However, evidence would suggest that the standard of care offered to people with learning disabilities and epilepsy is significantly poorer than in the general population, as is the case for most disease areas affecting people with learning disabilities[5,8].

In order to improve the management of epilepsy and the lives of those affected by both learning disabilities and epilepsy, improvements in education, communication and collaboration between patients, their carers and clinical teams are required[12]. The Books Beyond Words booklet: 'Getting on with Epilepsy' has been designed to help people with learning disabilities understand difficult issues and manage their health and illness through images that have all been tested with people with mild and severe learning disabilities (some examples of images are included in appendix 4). The booklet has been developed by the Royal College of Psychiatrists, and Books Beyond Words, with systematic input from people with learning disabilities, their carers, consultant psychiatrists, patient groups and other medical

professionals[15]. The intervention is based on the assumption that people with learning disabilities who cannot read and have limited communication abilities are visually literate. While the success and impact of the Book Beyond Words booklets (over 30 available) have been recognised by several awards and collaborations with established patient organisations, to date they have not been formally evaluated in clinical settings or routinely adopted in the NHS. The lack of conclusive data on the effectiveness of educational interventions for people with epilepsy and learning disabilities, as well as the current emphasis on empowering this population to better manage their condition and prevent premature avoidable deaths, warrant further investigation[8,31].

We will therefore undertake a randomised controlled feasibility trial of the Books Beyond Words booklet for epilepsy designed to improve the management of epilepsy for people with learning disability and their carers. The feasibility study will not demonstrate whether or not Books Beyond Words interventions can achieve those goals, and provide value for money, However, if feasibility is proven, we plan a further definitive trial to address effectiveness and cost effectiveness. If the intervention is effective (as demonstrated by the definitive trial), it will contribute to improve disease management of people with epilepsy and learning disability, increase quality of life, knowledge and in turn improve the standard of care and access to relevant services. Improving knowledge, disease management and access to services of people with learning disabilities and epilepsy should also reduce costs and benefit the wider population.

3.2. Aims and objectives

Our overall aim is to conduct a randomised controlled feasibility trial exploring key methodological, design and acceptability issues, in order to subsequently undertake a large-scale randomised controlled trial of the Book Beyond Words booklet for Epilepsy as an intervention for epilepsy in patients with learning disabilities.

The following research questions will guide the study design, data collection and analysis:

- What is the feasibility of undertaking a randomised controlled trial of the Books Beyond Words intervention for epilepsy in people with learning disabilities?
- What is the potential effectiveness and cost effectiveness of this intervention?
- Is this intervention acceptable to patients with learning disabilities, carers and health professionals?

Our objectives will be to:

<u>Quantitative Assessment</u>

- Evaluate the number of patients screened, the proportion of eligible patients, and the proportion of recruited patients across both intervention and control arms;
- Evaluate the proportion of patients who refused to take part in the trial and the reasons given;
- Evaluate the number of recruited patients who completed the questionnaires;
- Assess the variability of the primary outcome measure (quality of life) for the treatment and control arms, allowing an assessment of effect size in order to inform the design of a definitive trial;
- Measure discontinuation rates across both study arms, the stages of discontinuation and associated reasons;
- Monitor the intervention’s patterns of use post randomisation, and explore whether other education/information resources on epilepsy have been used;
- Explore the feasibility of collecting health resource use and quality of life data, to inform the design of the health economics component of a future definitive trial.

Qualitative Assessment

- Explore the views of patients, their carers and health professionals regarding the facilitators and barriers to participating in this trial, such as recruiting, randomisation, completing questionnaires and the relevance of the questions;
- Explore the acceptability of the Books Beyond Words booklet among patients with learning disabilities, their carers and health professionals;
- Explore potential weaknesses of the study design (e.g. the consistent and adequate use of the booklet in the intervention group; impact of posting questionnaires on completion and discontinuation rates etc).

4. Methods

4.1. Design

We will use a two-arm, single-centre design and will conduct the study over a 20-month period.

4.2. Setting

The study will be conducted in a single NHS trust: Hertfordshire Partnership NHS Foundation Trust. The trust runs 5 Epilepsy Clinics, managing 196 patients with learning disabilities and epilepsy. The patients are seen once a month. The clinics are held, in the following locations:

- Rosanne House, Parkway, Welwyn Garden City, AL8 6HG;
- Ware Road Day centre in Hertford;
- Lister Hospital, in Stevenage;
- The Orchards in Hemel Hempstead;
- Albany Lodge in St Albans.

4.3. Participants

Participants will be recruited across five Epilepsy Clinics, over 6 months. Eligible participants will be identified by the Consultant Psychiatrists who run the Epilepsy Clinics listed above. A log of potentially eligible patients will be compiled by each psychiatrist from existing patient records and from new referrals using the criteria below.

Inclusion criteria:

- Male and female patients, over 18 years of age;
- A confirmed clinical diagnosis of epilepsy (according to medical notes) and at least one seizure over the past 12 months;
- A confirmed clinical diagnosis of a learning disability (significantly below-average general intellectual functioning and an IQ below or equal to 70);
- Ability to communicate verbally. The patient has a vocabulary of more than 10 words and can use 1 to 2 words, or more, to communicate;
- The carer is sufficiently proficient in English to read and complete the questionnaires with the patient.

4.4. Study flow-chart

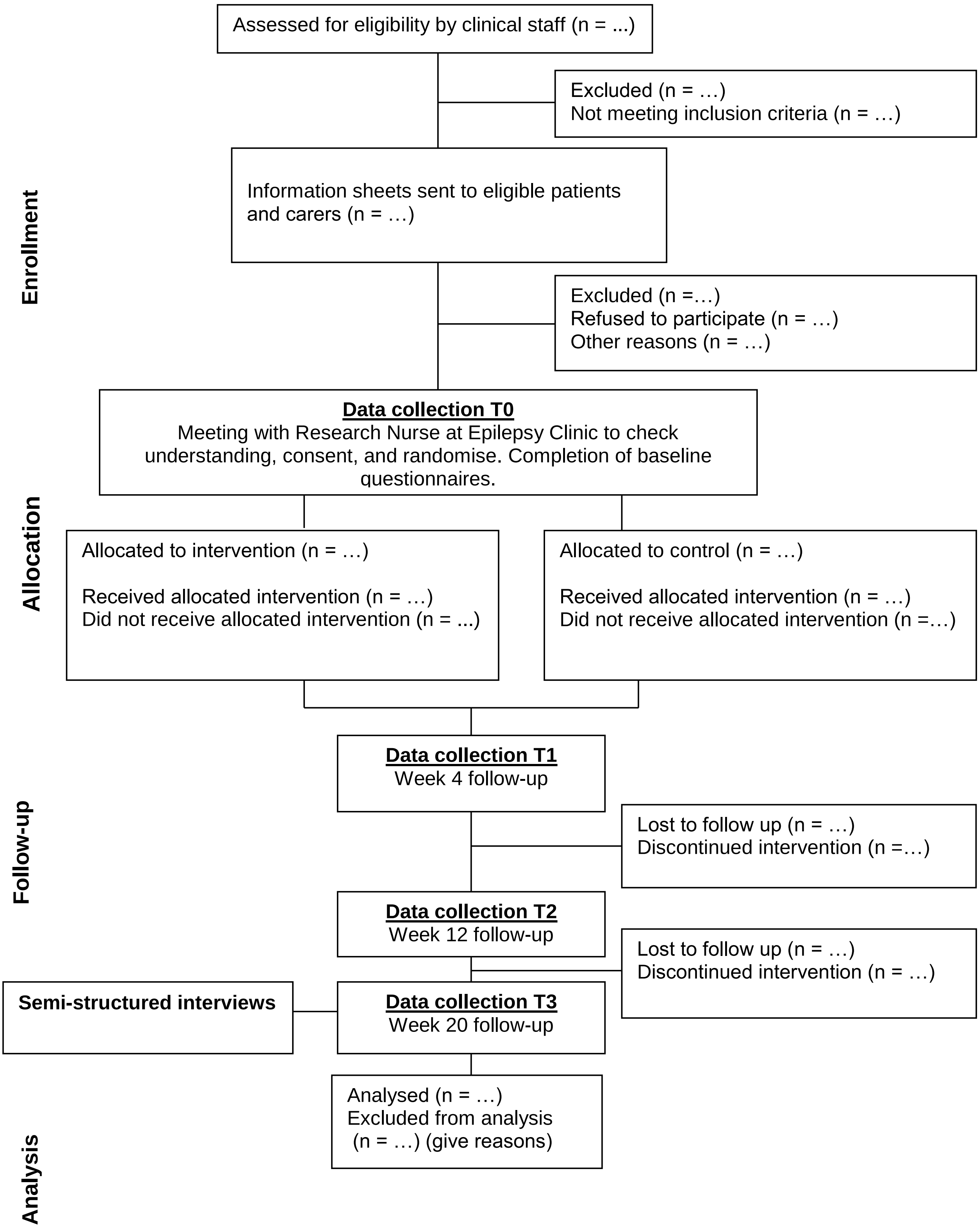

Exclusion criteria:

- Vision impairment;
- Confirmed diagnosis of dementia;
- Has used the Books Beyond Words booklet for epilepsy in the past 12 months.

4.5. Intervention

The Books Beyond Words booklet: 'Getting on with Epilepsy', uses pictures to tell a story about a young man with learning disabilities and epilepsy who progressively learns how to better manage epilepsy and recurrent seizures (see appendix 4). All images have been tested with people with mild and severe learning disabilities to ensure understanding. The intervention aims to improve seizure control, reduce the risk of falls and seizure-related injuries, and improve quality of life. It also aims to illustrate best practice and promote access to relevant services. The Books Beyond Words booklet for Epilepsy was developed over an 18-month period, by an Editorial Team of Consultants in Psychiatry and Neurodevelopmental Psychiatry, a Speech and Language Therapist and an Illustrator. They were supported by an advisory group of medical experts in the field of epilepsy and learning disabilities, by patients with learning disabilities and their carers, voluntary organisations and advocacy groups. Several iterations were developed and tested with a wide group of stakeholders including patients with mild to severe learning disabilities and epilepsy[15].

4.5.1. How the Books Beyond Words intervention will be used

Health professionals and carers can create their own story or use the story provided at the end of the booklet. The black and white pictures are for use by health professionals only, to discuss the clinical management of epilepsy with the patient. The booklet contains instructions for use and a list of useful resources and services. In the proposed study, as recommend by Books Beyond Words, the booklet will be used as follows:

1) First, the Research Nurse looks at the pictures before using the book with the patient and carer.

2) The Research Nurse shows the first picture of the booklet to the person with learning disabilities. He/she should be encouraged to hold the book and turn the pages at his/her own pace.

3) Depending on the communication abilities and reactions of the patient, it is advised that the Research Nurse prompts him/her to say what is happening, whether this has happened to

him/her before, and how he/she feels about it.

4) It is not always possible to look at the booklet in one sitting. The Research Nurse will always follow the patient's own pace. If the patient loses interest and the book cannot be explored in one session, the Research Nurse will schedule a second session.

5) Finally, the Research Nurse will also use and photocopy the black and white images provided at the end of the booklet to create personalised advice and epilepsy related health promotion materials.

4.5.2. Training

Training in using the intervention (half-day training session delivered at Hertfordshire Partnership NHS Foundation Trust by Books Beyond Words) will be provided to the Research Nurse and Consultant Psychiatrists running the Epilepsy Clinics. An online video and training slides provided by Books Beyond Words will be made available to all carers, explaining how to use the booklet with the patient. Written instructions detailing how to use the intervention are also included in the booklet.

4.6. Control Group

Participants in the control group will receive routine information and services. The use of a picture book such as the Books Beyond Word intervention or other educational package will not be part of the routine consultation. After the study has terminated, patients in the control condition will be provided with a Books Beyond Words booklet to use with the Epilepsy Nurse Specialist, the consultant and/or the carer (see 4.8 Procedure for further information).

4.7. Outcomes

Each patient will be assessed at baseline (T0), and at 4 (T1), 12 (T2) and 20 (T3) weeks after randomisation (see study flow-chart). All questionnaires will be completed by the carer, with the patient's involvement, whenever possible. The baseline questionnaire will be completed at the initial meeting with the Research Nurse. The follow-up questionnaires will be sent to the carer in the post and returned in a prepaid envelope (see procedure for further information).

4.7.1. Quantitative assessment

The following outcomes will be measured (see Table 1):

- Number of eligible patients and number recruited:

The number of patients screened, proportion eligible, and proportion consented and recruited will be collected on the screening log. A ratio will then be calculated.

- Variability of the primary outcome quality of life:

We will use the Epilepsy and Learning Disabilities Quality of Life (ELDQOL) scale[33,34]. This scale consists of four subscales (behaviour, seizure severity, mood, and side-effects) and there is good evidence of the reliability and validity of this scale[34]. This scale will be completed by the carer with the patient's involvement, at T0, T1, T2, and T3.

- Discontinuation rates across both arms and reasons:

We will monitor both withdrawals from the study and discontinuation of the intervention (i.e. use of the booklets). These rates will be estimated on the basis of a Case Report Form completed by the nurse and return of postal questionnaires.

- Patterns of use of the Books Beyond Words booklet (for intervention group only):

The Research Nurse will record how many booklets have been provided to the patient and carer (2 booklets maximum) on the Case Report Form (see appendix 5). At T1, T2, and T3, carers will be asked how many times the booklet has been used, for how long, and with whom. These questions will be part of the questionnaire sent to carers in the post (see appendix 6-12)

- Use of other epilepsy related information:

At T0 and T3, carers and patients in the intervention and control groups will be asked which other epilepsy education or information resources (if any) have been used.

- Resource use and cost-effectiveness:

We will assess levels of resource-use associated with the epilepsy booklet and the use of other NHS and Personal Social Services (PSS) at T0 and T3 (see questionnaires in appendix 6-12). The EQ-5D[35] will be used to measure quality of life at T0, T1, T2, and T3. This measure consists of five dimensions (mobility, self-care, usual activities, pain/discomfort and anxiety/depression), and a visual analogue scale to record the respondent's self-rated health.

- Demographic data:

We will collect the following demographic information at baseline, about both the participant and the carer: age, sex, ethnicity, and living circumstances of the participant, information about the type of care provided by the carer and relationship to the participant (see baseline questionnaire in appendix 6). All information will be provided by the carer, with the participant's involvement whenever possible.

4.7.2. Qualitative assessment

We will conduct semi-structured qualitative interviews with a random sample of 15 patients and carers (or until data saturation) selected from both control and intervention groups, as well as health professionals involved in the feasibility trial. They will be interviewed by the Research Fellow at 20 weeks post randomisation at the five Epilepsy Clinics (see information sheet and consent form for health professionals and interview schedules in appendix 13-16).

The semi-structured interviews will investigate:

- Feasibility and acceptability of the recruitment, allocation, randomisation and data collection procedures;
- Use and perceived usefulness of current information/education materials and services available;
- Information and self-management support needs;
- Perceived acceptability of the Books Beyond Words booklet;
- Potential weaknesses of the study design (adequate use of the booklet, use of postal questionnaires etc);
- Perceived barriers and facilitators to the use and dissemination of the intervention in routine care.

Table 1: Outcome measures according to study schedule

**Measure**	T0 baseline assessment	T1 week 4	T2 week12	T3 week 20
**Rates of recruitment**	X	X	X	X
**Quality of Life** (ELDQOL scale)	X	X	X	X
**Discontinuation rates**	X	X	X	X
**Intervention's patterns of use**	X	X	X	X
**Demographic data**	X			
**Seizure severity** (using ELDQOL Seizure Severity Scale)	X			
**Seizure control** (diary cards; see appendix 17)	X	X	X	X
**Feasibility and acceptability** (qualitative interviews)				X
**Resource use**	X			X
**Quality of Life** (EQ-5D-5L scale)	X	X	X	X

4.8. Procedure

4.8.1. Trial Setup

This will involve obtaining ethical approval from the NHS Research Ethics Committee, R&D permission, registration on the CLRN portfolio, data management set-up and Book Beyond Words training before the study start date. Other tasks will include recruiting the Research Fellow and Research Nurse, undergoing GCP training whenever relevant, establishing the Trial Steering Committee and Reference Group.

4.8.2. Screening

Prior to the study start date, Consultant Psychiatrists in Learning Disabilities and an Epilepsy Nurse Specialist will perform a computerised search to screen for eligible patients among those already registered at the Trust as well as recent referrals. Eligible patients will be entered into the screening/randomisation log.

4.8.3. Consent and baseline assessment

Each Consultant will send an invitation letter and two information sheets to the carers of all eligible patients with learning disabilities and epilepsy (see appendix 18-21). In accordance with the Mental Capacity Act, an easy read information sheet will be intended for the patient (who is also the study participant) and the other for the carer. The easy read patient information sheet and consent form have been designed according to the principles of the Mental Capacity Act, guidance from the Department of Health on producing easy read information and guidance from the National Ethics Service on the design of study materials for adults without capacity (see information sheet and consent form in appendices 21-22). The information is paced, carefully structured, using simple language and images, with frequent references made to the carer. The consent form and information sheets have been reviewed by our reference group. It includes people with learning disabilities, carers, members of the Public Involvement in Research group (PIRG) and a representative from the Books Beyond Words association.

In the invitation letter, carers are asked to encourage patients who have the capacity to read and understand (or to be read and understand) the easy read information sheet to do so. For patients who lack the mental capacity to understand the information sheet, it is the carer's role to read the information sheet and use what they know of the patient's wishes and feelings about research to decide whether he/she would like to take part in the study. The researcher's contact details are provided in case the patient or carer have any questions while considering whether or not to take part in the study.

After a week, the Research Nurse will invite carers and patients to attend a meeting at the Epilepsy Clinic, at a time that is convenient for them. During this appointment, the Research Nurse will go through the Patient Information Sheets with both the patient and the carer, and will then take consent from the patient, with the carer's involvement and advice, using an easy read consent form. For patients who lack the capacity to consent, the Research Nurse will use a Consultee Declaration Form (see appendix 22-23). There will be three signed copies of the easy read consent form or consultee declaration form: one copy will be kept by the Research Team, one copy will be given to the participant and carer, and one copy will be kept in the medical notes. The Research Nurse will send a letter to the patient's GP (see appendix 24).

The carer will then be asked to complete the baseline questionnaire with the patient's involvement (see questionnaire in appendix 6). Once the baseline questionnaire has been completed, the Research Nurse will randomise the patient to the intervention or control arm. It is anticipated that the appointment will take approximately 40 minutes; 10 minutes for the information and consent procedure and 30 minutes to complete the baseline questionnaire.

4.8.4. Randomisation

Randomisation will be managed by the Data Management Team at the Norwich Clinical Trials Unit (CTU). Authorised users (Research Nurse) will login using their own unique Username and Password to a secure website to randomise each participant. The results will be stored in a study database on the Norwich CTU secure server at UEA (no user identifiable data will be stored in the randomisation database). Web traffic will be encrypted using standard secure sockets layer technology. The randomisation (1:1 allocation ratio) will be 'blocked' into groups of 6 codes to reduce the imbalance of Control and Intervention group participants at any time.

4.8.5. Intervention condition

After giving consent and completing the baseline questionnaires, patients in the intervention condition will use the booklet with the Research Nurse, and carer, in a quiet room at the Epilepsy Clinic. The nurse will go through the booklet with the patient, at the patient's own pace. If the booklet cannot be explored in one sitting, the Research Nurse will arrange another appointment. We anticipate that it can take up to one hour to go through the booklet. The patient will also be given a copy to take home and explore with the carer and/or relatives. It is recommended that the patient is given the opportunity to look at the book at least twice more at home. The carers will not receive any formal training in using the booklet but will be able to watch an online video and training slides explaining how to use the book with the person they care for. A link to the training video and slides will be provided by the Nurse at the end of the initial meeting. They would also have had the opportunity to see the Research Nurse use the booklet with the participant and ask any questions about future use.

The Research Nurse will call each carer two weeks after randomisation to check that they have been able to look at the booklet again. She will record all relevant information (including the number of sessions needed to go through the booklet) on the case report form (see appendix 5). Use of the book at home with the carer or other relatives will be monitored as part of the questionnaire completed by the carer at one month, three months, and five months post

randomisation (see appendix 7-12). Postal questionnaires will be sent to the carer and patients at 4, 12 and 20 weeks post randomisation. Given most patients will not have the capacity to read and complete questionnaires on their own, the questionnaires will be completed by the carer, who will systematically involve the patient in this process. The Research Fellow will undertake questionnaire follow-ups on the phone at 4, 12 and 20 weeks post-randomisation, to remind the carers to complete and return them.

4.8.6. Control condition

As mentioned in section 4.6, patients in the control condition will receive usual information and services. The use of picture book such as the Books Beyond Word intervention or other educational package will not be part of the routine consultation. We will monitor the use of other epilepsy related information/education materials in both groups. The questionnaires will be completed as stated above.

4.8.7. Qualitative interviews

A random sample of carers, patients and health professionals will be invited for a semi-structured interview at the Epilepsy Clinic. With the carer's involvement, participants' capacity to take part in an interview will be re-assessed. Interviews will be audio-recorded and are expected to take approximately 30 minutes. The Research Fellow will keep interviewing participants until data saturation has been reached.

4.8.8. Study termination

The study will end at week 20, and all participants will resume routine care. Patients in the control condition will be provided with a Books Beyond Words booklet to use with the Epilepsy Nurse Specialist, the consultant and/or the carer, after the study has terminated.

4.9. Proposed sample size

One of the objectives of this randomised controlled feasibility study is to assess variation in the primary outcome measure in order to determine whether the intervention is likely to achieve a 10% increase in the participants' quality of life[40]. This would equate to an effect size of approximately .4 requiring a total sample size of 174 in a full study (1-β=.8, α=0.05). This therefore indicates a required sample size of 16 per group (e.g. 9% per group), and allow

exclusion of an effect size of <0. Given a 25% drop out, a sample size of 20 per group (40 participants in total) will be targeted.

4.9.1. Feasibility of recruitment

Recent audit data indicates that there are 196 patients with epilepsy and learning disabilities registered at the Hertfordshire Partnership NHS Foundation Trust. A preliminary screening exercise has already been undertaken by the Lead Consultant Psychiatrist, at each Epilepsy Clinic, confirming that 112 out of 196 patients meet the study's inclusion criteria. This does not include new referrals, which will be screened for eligibility once recruitment starts.

4.10. Statistical analysis

4.10.1. Quantitative assessment

The analysis primarily aims to determine the feasibility of undertaking a full-scale randomised controlled trial. The analysis will seek to assess:

1) The number and demographics of eligible patients identified, screened, consented and randomised to each arm, and the relative performance in each Clinic;

2) The demographics of patients who are excluded from the trial, reasons for exclusion, and estimation of the proportion of eligible patients approached who agree to enter and complete the trial;

3) The proportion of completed study measures in each arm, measured from baseline to the last follow-up assessment;

4) The proportion of patients withdrawing from the study, measured from baseline to the last follow-up assessment;

5) The variation in the primary outcome measure that will be used, following Cocks and Torgerson[40], to determine whether a one-sided 80% confidence interval would exclude a 10% increase in the primary outcome (ELDQOL);

6) Describe the frequency of use of the Books Beyond Words intervention, and other sources of information before and after randomisation, and determine the extent to which use of the Books Beyond Words influences outcome and other information resource use;

The analysis will be reported according to relevant CONSORT standards[41].

4.10.2. Qualitative assessment

The data collected in the semi-structured interviews will be analysed using a two-step thematic content analysis derived from descriptive phenomenology[42-44], assisted by the computer software ATLAS-ti.

4.11. Health economic analysis

Estimation of cost-effectiveness, within a health-technology assessment, is an iterative process[45]. Here we aim to monitor levels of resource-use and quality of life, to inform the decision as to how costs and benefits should be measured as part of a future, more definitive study. NICE guidance recommends that costs are calculated from the perspective of the NHS and Personal Social Services (PSS)[46]. We will thereby seek to monitor levels of resource-use associated with the epilepsy booklet and the use of other NHS and PSS resource-items. Appropriate unit costs will subsequently be attached to all items of resource-use, to estimate the mean overall cost in each study-arm[47]. In line with NICE guidance, the EQ-5D[35] will be used to assess the effectiveness of each option (this will enable the QALY (Quality Adjusted Life Year) gain to be estimated). The main purpose of the analysis is to inform how the above data on costs and effects would be collected within a more definitive study. Thus, we will estimate completion rates and seek to identify big cost drivers, in order to inform this decision. Additionally, though the results of this will need to be treated with caution, a preliminary cost-effectiveness analysis will also be performed.

4.12. Data management

Data management will be provided by Norwich Clinical Trials Unit and will include provision of a web-based data entry system – the data will be stored in a database on a server at Norwich CTU, at UEA. The server is in a secure room and access is restricted to UEA IS and NCRTU Data management staff. The study database will be built using Microsoft SQL Server tools and direct access will be restricted to CRTU IS staff. Data entry will be via web pages created using Microsoft.NET technology. Access will be by dedicated user name and password for study team members (Research Nurse, Researcher etc) and CRTU Data management staff only. All internet traffic will be encrypted using the standard SSL (Secure Sockets Layer) methodology. The data entry system will validate data on entry to ensure it is of the expected type (e.g. integers, dates etc.) and range of values. Periodically and at database lock, the data will be further validated for errors and inconsistencies.

The database is linked to an audit tool where all data additions, modifications and deletions are recorded with date/time and the user ID of the person making the change. The database is designed to comply with the ICH Guideline for Good Clinical Practice (GCP), within the Standard Operating Procedures for Data Management in NRCTU and also where appropriate with UEA IT procedures. NCRTU will provide data validation and dataset creation at the end of the study for analysis. The randomisation will be included as an integral part of the system and the results will be displayed on screen and/or emailed to nominated individuals.

4.13. Write-up and dissemination

The results of this randomised controlled feasibility study will be used to inform the design of a full-scale randomised controlled trial of the Books Beyond Words booklet for Epilepsy. Should the findings confirm feasibility, a funding application will be submitted to the Clinical Evaluation and Trial funding scheme, part of the National Institute for Health Research, Health Technology Assessment Programme. Furthermore, the results of the feasibility trial will be of utmost importance to patient groups and clinicians, in raising awareness of emerging research in learning disabilities and epilepsy and demonstrating the potential acceptability of this intervention. Dissemination of the results will primarily be achieved through publication of the findings in peer-reviewed scientific journals, and in a report intended for people with learning disability, their carers, families and the wider lay community, disseminated through the Making it Better Group, Inclusion East, and the British Institute of Learning Disabilities (BILD). BILD will also assist with dissemination through its newsletters circulated to over 6000 stakeholders. The study findings will also be showcased at one of their annual conferences. Further dissemination will be carried out by several of the project's co-applicants (Professor Bob Gates, Dr Howard Ring and Dr Marie-Anne Durand) who have longstanding track records of lecturing on learning disabilities and epilepsy, of running workshops and regularly talking to local and national patient groups, as well as attending national and international scientific conferences. The findings from the feasibility study will be presented as oral and poster presentations at major national and international conferences. Finally, research among patients with learning disabilities is uncommon. Promoting the involvement of this population in research and demonstrating potential benefits will be another focus of our dissemination strategy. We will work with the BILD and with the Patient Involvement in Research Group (PIRG) to raise awareness and increase the representation of patients with learning disabilities and their carers in research.

5. Timeline

The study includes 4 phases (see timetable in appendix 25):

Phase 1: Trial set-up (months 0-2) deliverables:

a) Recruitment of research and clinical staff to establish the trial team.

b) Trial documents including final protocol, patient information sheets, consent forms and questionnaires submitted for ethical approval to the NHS Research Ethics Committee.

c) Research and Development approval from Hertfordshire Partnership University NHS Foundation Trust.

d) Study adopted on the CLRN portfolio.

e) Books Beyond Words training delivered to the Research Nurse and Consultant Psychiatrists by Books Beyond Words.

f) GCP training.

Phase 2: Recruitment and randomisation (months 2-8) deliverables:

a) 112 subjects contacted (+ new referrals); 40 consented, recruited and randomised.

b) Baseline questionnaires completed.

Phase 3: Quantitative and qualitative assessments (months 3-13) deliverables:

a) Follow-up questionnaires (at 4, 12 and 20 weeks) completed.

b) Qualitative interviews undertaken at 20 weeks.

Phase 4: Data analysis, write-up and dissemination (months 11-20) deliverables:

a) Qualitative data analysis completed.

b) Statistical analysis and health economic modelling completed.

c) Report on trial outcomes written-up.

d) Abstract submission for conference posters/oral presentation.

e) First draft of journal manuscript.

f) Application for full-scale trial funding.

6. Ethical considerations

6.1. Consent

One week after receiving the information sheet (see section 4.8.3 for further information) carers and patients will be invited to attend an appointment with the Research Nurse, at the Epilepsy Clinic. The Research Nurse will go through the Patient Information Sheets with both the patient and carer. The Research Nurse will then take consent from the patient, with the carer's involvement and advice, who will assess the participant's capacity to take part, as specified in the Mental Capacity Act. Easy read consent forms will be used with participants who have the capacity to read and understand (or be read and understand) simple information about the study. The information is paced, carefully structured, using simple language and images with frequent references made to the carer. For participants who lack the capacity to consent, a consultee declaration form will be completed by the carer. The carer will consider what he/she knows of the patient's wishes and feelings about research to decide whether or not to take part. The consent form has been reviewed by our reference group, which includes people with learning disabilities, carers, members of the Public Involvement in Research group (PIRG) and a representation from the Books Beyond Words association.

6.2. Data protection and confidentiality

This study will be conducted in accordance with the Data Protection Act 1998[1] and the guidelines of the Declaration of Helsinki 1964 (updated Tokyo 2004)[3]. Access to the screening/randomisation log, containing patient identifiable data, will be restricted to authorized personnel and will be password protected. Only GCP certified investigators approved by the Trial Steering Committee will be given access to the data. A unique Patient Identification Number (PID) will be generated by the study web-based data entry system for each new patient entered by the Research Nurse in the system. A sequential number starting at 001 will be generated for each new patient consented and entered into the study by the Research Nurse.

The original signed consent forms will be retained at the study site, in a locked cabinet. After all participants have been recruited, the researcher will collect baseline questionnaires from the Epilepsy Clinics. All case report forms and signed consent forms will be collected at the end of the study and stored in a locked cabinet at the University of

Hertfordshire. Patient data relating to the study will be deleted or destroyed within 3 months of the end of the study. Anonymised study data will be archived by the University of Hertfordshire for 5 years after study completion in line with standard research protocols. Investigators will not disclose patient data in any form to anyone not involved directly in the study. All electronic data will be stored on password-protected computers, and paper files will be stored in locked filing cabinets; both of which will be kept within electronically locked offices.

6.3. Other ethical considerations

It is very unlikely that participants will experience adverse events or will be distressed as a result of taking part in this study.

7. Expected output and impact of research

The outputs of this feasibility study will be used to inform the design and methodology of a definitive study, adequately powered to determine the impact of the Books Beyond Words intervention to improve self-management of epilepsy in people with learning disabilities. Should the present study indicate that a definitive study is feasible, a funding application will be submitted to the Clinical Evaluation and Trial funding scheme, part of the National Institute for Health Research. NICE advises that people with epilepsy and learning disabilities are empowered to improve disease management and outcomes, which should be achieved by providing adapted information and education about epilepsy and the same standard of care, specialist epilepsy services and investigations as the general population. This is exactly what the Books Beyond Words intervention aims to accomplish. Improving knowledge, disease management and access to services of people with learning disabilities and epilepsy should also reduce costs. Although the feasibility study will not demonstrate whether or not Books Beyond Words interventions can achieve those goals, and provide value for money, this will be addressed by the subsequent randomised controlled trial. The results of the definitive trial will therefore have significant and long lasting implications for clinical practice and service delivery, whereby Books Beyond Words interventions could be available and implemented in the NHS, for a variety of topics and conditions, including epilepsy, yielding significant benefits for patients and their carers.

8. Sponsorship and indemnity

8.1. Sponsor's details

The study is to be sponsored by Hertfordshire Partnership University NHS Foundation Trust.

The address of the sponsor is:

Hertfordshire Partnership University NHS Foundation Trust,

99 Waverley Rd,

St Albans,

Hertfordshire AL3 5TL.

Tel: 01727 804700.

Contact: Chief Investigator, Professor Bob Gates.

Details of the sponsor's responsibilities can be found in appendix 1.

8.2. Indemnity arrangements

The study will be covered under Hertfordshire Partnership University NHS Foundation Trust's indemnity policy. Details of indemnity arrangements can be found in appendix 1.

9. Study Management and finance

9.1. Study management

The Chief Investigator, Professor Bob Gates, will be accountable to the Sponsor and will hold overall responsibility for the trial, including submission of required progress-reports to the NIHR, and NHS ethics committee, deliverables, financial statements and the correct use of funds. Dr Marie-Anne Durand will coordinate the study set-up, including ethical approval, staff training as well as all operational issues arising at the Epilepsy Clinics. She will work in close collaboration with the other Co-Applicants, trial staff, the Clinical Trial Support Network (CTSN) at the University of Hertfordshire, and the Clinical Trials Unit at the University of East Anglia. Regular ad hoc meetings will be held to monitor day-to-day progress, and address relevant issues that arise. The Trial Coordinator will report to the Trial Steering Committee (TSC).

9.2. Trial Steering Committee

The ongoing management of the study will be conducted through regular meeting of the Trial Steering Committee (TSC). The TSC will meet five times (see timetable in appendix 25) and will be chaired by an independent person with relevant trial experience (TBC). It will include the Chief Investigator, Co-Applicants, Mrs Lesley Barcham (from BILD), and another PIRG member. The TSC will identify and resolve study-related problems, taking early action if recruitment, or other aspects of the study, go off-target.

9.3. Reference group

Patient and public involvement will be central to the design and management of the research, the development of patient information and consent materials, the reporting of the study results and dissemination. This will ensure accessibility, acceptability and usability of the study procedures and materials, and guarantee that the needs and potential concerns of people with learning disabilities and epilepsy and of the wider public are represented and accounted for. Their involvement will also maximise the applicability of the findings and facilitate the dissemination of the study results through patient groups, and the UK charity: BILD.
A reference group comprising eight patients, carers and members of the public will be convened, and will meet on five occasions over 20 months, in parallel to the TSC (see Gantt Chart in appendix 25). The reference group will include members of the 'Making it Better Group' which includes patients with learning disabilities and carers, members of 'Inclusion East', comprising patients and families, and two members of the PIRG group. Members of the

reference group will be invited to review study materials, progress reports, and other relevant study documentation, and will discuss potential challenges, and advise on necessary changes and next steps. Their comments and discussions will be fed back to the TSC. The reference group will be supported by the PIRG: an effective and long-standing mechanism for involving the public in research, developed by the Centre for Research in Primary and Community Care (CRIPACC) at the University of Hertfordshire. The PIRG members have been involved in various research projects and have developed the skills required to support and empower others in taking part in research projects. They will act as champions within the reference groups, and will provide support and training to others, as and when necessary.

9.4. Financial responsibility and management

This study has been awarded a grant of £200,999, to be transferred to the lead NHS partner (Hertfordshire Partnership University NHS Foundation Trust) upon receipt of ethical approval and the study start date. £141,703 will then be paid to the University of Hertfordshire for study costs.

The budget for the NHS will be held by the Signatory at the Trust: Professor Tim Gale and Professor Bob Gates, the Chief Investigator.

The budget for UH will be held by Dr Marie-Anne Durand. Financial decisions will be shared, whenever possible, with the TSC.

10. References

1. Great Britain Parliament. Data Protection Act: chapter 29. In. London: Stationery Office; 1998.
2. Great Britain Parliament. Mental Capacity Act. In. London: The Stationery Office; 2005.
3. 55th WMA General Assembly. Declaration of Helsinki 1964 (updated Tokyo 2004). In. Tokyo: World Medical Organization; 2004.
4. van Blarikom W, Tan IY, Aldenkamp AP, van Gennep ATG. Epilepsy, intellectual disability, and living environment: A critical review. Epilepsy & Behavior 2006;9:14-8.
5. Emerson E, Baines S, Allerton L, Welch V. Health Inequalities and People with Learning Disabilities in the UK; 2011.
6. Pennington M, Prince E, Bateman N, et al. Factors influencing the costs of epilepsy in adults with an intellectual disability. Seizure 2012;21:205-10.
7. van Schrojenstein Lantman-de Valk HM, Walsh PN. Managing health problems in people with intellectual disabilities. BMJ 2008;337:a2507.
8. Improving Health and Lives: Learning Disabilities Observatory. Improving the health and wellbeing of people with learning disabilities: An evidence-based commissioning guide for clinical commissioning groups (CCGs); 2012.
9. McGrother CW, Bhaumik S, Thorp CF, Hauck A, Branford D, Watson JM. Epilepsy in adults with intellectual disabilities: prevalence, associations and service implications. Seizure 2006;15:376-86.
10. Morgan CL, Baxter H, Kerr MP. Prevalence of epilepsy and associated health service utilization and mortality among patients with intellectual disability. Am J Ment Retard 2003;108:293-300.
11. Brodie MJ, Dichter MA. Antiepileptic drugs. N Engl J Med 1996;334:168-75.
12. Hannah JA, Brodie MJ. Epilepsy and learning disabilities--a challenge for the next millennium? Seizure 1998;7:3-13.
13. Kerr M. Epilepsy and learning disabilities. In: Epilepsy ILAEUCaNSf, ed. Epilepsy: from cell to community, a practical guide to epilepsy. 11th edition ed. London; 2007.
14. Clark AJ, Espie CA, Paul A. Adults with learning disabilities and epilepsy: knowledge about epilepsy before and after an educational package. Seizure 2001;10:492-9.
15. Books Beyond Words website. (Accessed 14 May, 2013, at http://www.booksbeyondwords.co.uk/.)
16. Mariani E, Ferini-Strambi L, Sala M, Erminio C, Smirne S. Epilepsy in institutionalized patients with encephalopathy: clinical aspects and nosological considerations. Am J Ment Retard 1993;98 Suppl:27-33.
17. van Schrojenstein Lantman-De Valk HM, Metsemakers JF, Haveman MJ, Crebolder HF. Health problems in people with intellectual disability in general practice: a comparative study. Fam Pract 2000;17:405-7.
18. van Schrojenstein Lantman-de Valk HM, van den Akker M, Maaskant MA, et al. Prevalence and incidence of health problems in people with intellectual disability. J Intellect Disabil Res 1997;41 (Pt 1):42-51.
19. Bowley C, Kerr M. Epilepsy and intellectual disability. J Intellect Disabil Res 2000;44 (Pt 5):529-43.
20. McDermott S, Moran R, Platt T, Wood H, Isaac T, Dasari S. Prevalence of epilepsy in adults with mental retardation and related disabilities in primary care. Am J Ment Retard 2005;110:48-56.
21. Chadwick D. Epilepsy. J Neurol Neurosurg Psychiatry 1994;57:264-77.

22. Beavis J, Kerr M, Marson AG. Pharmacological interventions for epilepsy in people with intellectual disabilities. Cochrane Database Syst Rev 2007:CD005399.
23. Branford D, Bhaumik S, Duncan F. Epilepsy in adults with learning disabilities. Seizure 1998;7:473-7.
24. Branford D, Bhaumik S, Duncan F, Collacott RA. A follow-up study of adults with learning disabilities and epilepsy. Seizure 1998;7:469-72.
25. Brodie MJ. Antiepileptic drugs, clinical trials, and the marketplace. Lancet 1996;347:777-9.
26. Clinical guidelines for the management of epilepsy in adults with an intellectual disability. Seizure 2001;10:401-9.
27. Kerr M, Bowley C. Multidisciplinary and multiagency contributions to care for those with learning disability who have epilepsy. Epilepsia 2001;42 Suppl 1:55-6; discussion 7-8.
28. Kerr M, Bowley C. Evidence-based prescribing in adults with learning disability and epilepsy. Epilepsia 2001;42 Suppl 1:44-5; discussion 50-1.
29. Glover G, Ayub M. How do people with learning disabilities die.; 2010.
30. Cockerell OC, Hart YM, Sander JW, Shorvon SD. The cost of epilepsy in the United Kingdom: an estimation based on the results of two population-based studies. Epilepsy Res 1994;18:249-60.
31. National Institute for Health and Care Excellence. The epilepsies: the diagnosis and management of the epilepsies in adults and children in primary and secondary care. NICE clinical guideline 137.; 2012.
32. National Institute for Health and Care Excellence. Managing epilepsy in children, young people and adults. NICE Pathways. ; 2013.
33. Baker GA, Jacoby A, Smith DF, Dewey ME, Chadwick DW. Development of a novel scale to assess life fulfillment as part of the further refinement of a quality-of-life model for epilepsy. Epilepsia 1994;35:591-6.
34. Buck D, Smith M, Appleton R, Baker GA, Jacoby A. The development and validation of the Epilepsy and Learning Disabilities Quality of Life (ELDQOL) scale. Epilepsy Behav 2007;10:38-43.
35. Brooks R. EuroQol: the current state of play. Health Policy 1996;37:53-72.
36. Duncan JS, Sander JW. The Chalfont Seizure Severity Scale. J Neurol Neurosurg Psychiatry 1991;54:873-6.
37. Jarvie S, Espie CA, Brodie MJ. The development of a questionnaire to assess knowledge of epilepsy: 2--Knowledge of own condition. Seizure 1993;2:187-93.
38. Espie CA, Watkins J, Duncan R, et al. Development and validation of the Glasgow Epilepsy Outcome Scale (GEOS): a new instrument for measuring concerns about epilepsy in people with mental retardation. Epilepsia 2001;42:1043-51.
39. Espie CA, Watkins J, Duncan R, et al. Perspectives on epilepsy in people with intellectual disabilities: comparison of family carer, staff carer and clinician score profiles on the Glasgow Epilepsy Outcome Scale (GEOS). Seizure 2003;12:195-202.
40. Cocks K, Torgerson DJ. Sample size calculations for pilot randomized trials: a confidence interval approach. J Clin Epidemiol 2013;66:197-201.
41. Schulz KF, Altman DG, Moher D. CONSORT 2010 statement: updated guidelines for reporting parallel group randomised trials. BMJ 2010;340:c332.
42. Denzin N.K., Y.S. L. Handbook of Qualitative Research. . Second ed. Thousand Oaks: Sage Publications; 2000.
43. Holloway I. Qualitative research in health care. Maidenhead: Open University Press; 2005.
44. Pope C, Ziebland S, Mays N. Qualitative research in health care. Analysing qualitative data. BMJ 2000;320:114-6.

45. Sculpher M, Drummond M, Buxton M. The iterative use of economic evaluation as part of the process of health technology assessment. J Health Serv Res Policy 1997;2:26-30.
46. National Institute for Health and Clinical Excellence. NICE Guide to the Methods of Technology Appraisal; 2008.
47. Curtis L. Unit costs of health and social care 2011. : Personal Social Services Research Unit, The University of Kent. ; 2011.

11. Abbreviations

BILD	British Institute of Learning Disabilities
CLiCIR	Centre for Lifespan and Chronic Illness Research
CONSORT	Consolidated Standards of Reporting Trials
CRIPACC	Centre for Research in Primary and Community Care
CTU	Clinical Trial Unit
ELDQOL	Epilepsy and Learning Disabilities Quality of Life
GCP	Good Clinical Practice
HPUT	Hertfordshire Partnership University NHS Foundation Trust
NIHR	National Institute for Health Research
PIRG	Public Involvement in Research Group
PSS	Personal Social Services
RfPB	Research for Patient Benefit
TSC	Trial Steering Committee
UEA	University of East Anglia
UH	University of Hertfordshire

12. Appendices

12.1. Collaboration agreement

Collaboration agreement V1.0 26.0

12.2. RfPB funding confirmation

RfPB funding confirmation email.docx

12.3. RfPB reviewer comments

RfPB Competition 21 - Summary of review

12.4. Books Beyond Words

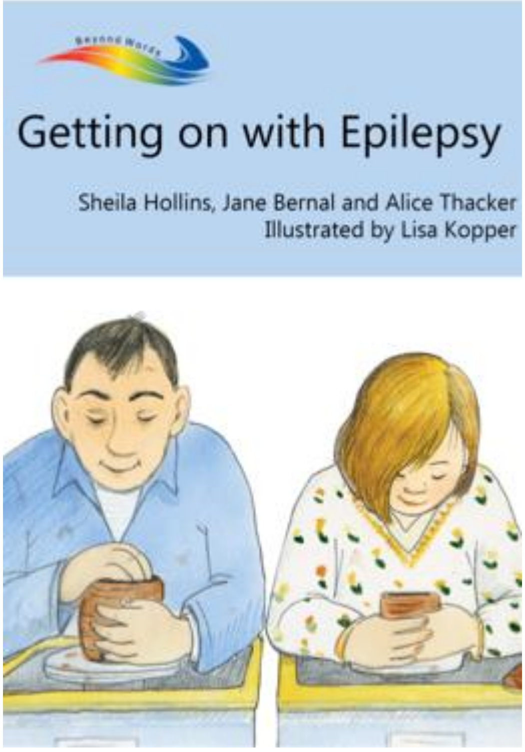

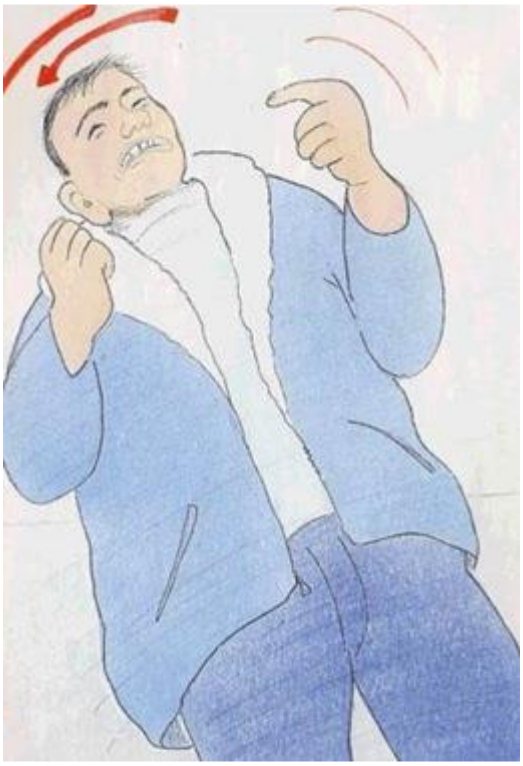

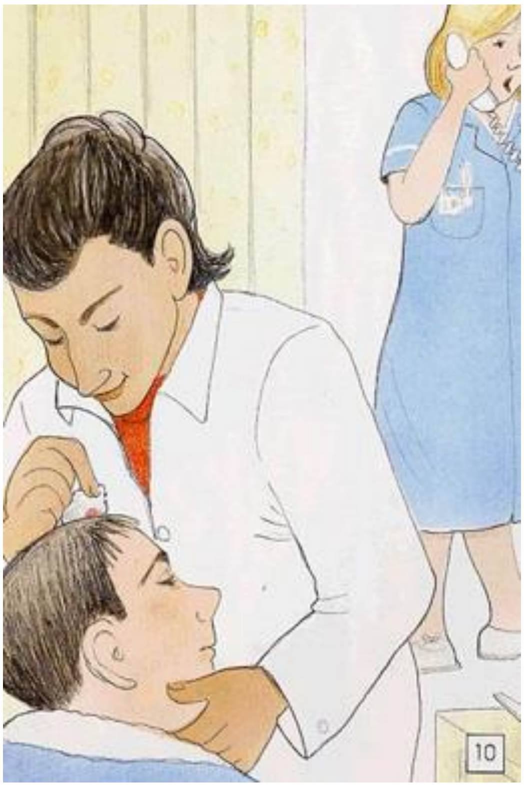

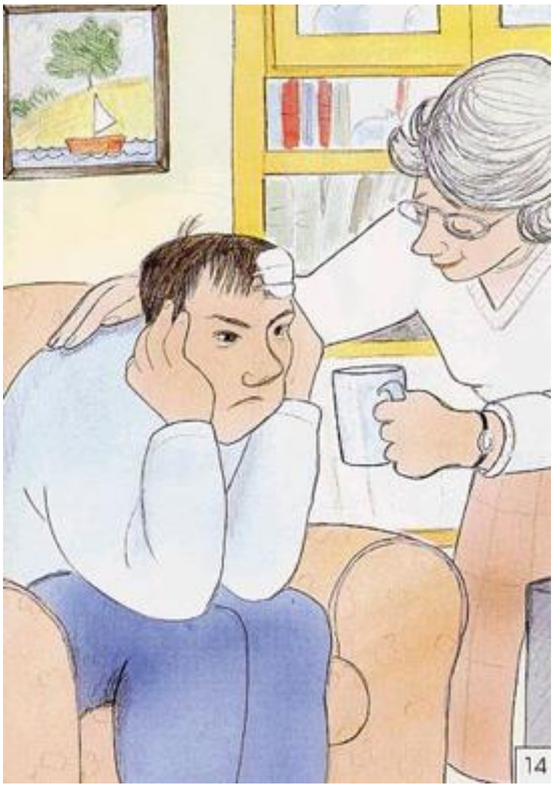

12.5. Case Report Form

Case Report Form V1.0.docx

12.6. Questionnaire baseline

Questionnaire V 1.0 baseline.docx

12.7. Questionnaire 4 week follow-up Intervention Group

Questionnaire V 1.0 - 4 week follow-up

12.8. Questionnaire 4 week follow-up Control Group

Questionnaire V 1.0 - 4 week follow-up

12.9. Questionnaire 12 week follow-up Intervention Group

Questionnaire V 1.0 - 12 week follow-up

12.10. Questionnaire 12 week follow-up Control Group

Questionnaire V 1.0 - 12 week follow-up

12.11. Questionnaire 20 week follow-up Intervention Group

Questionnaire V 1.0 20 week follow-up I

12.12. Questionnaire 20 week follow-up Control Group

Questionnaire V 1.0 20 week follow-up C

12.13. Health Professional Information Sheet

Health Professional Information Sheet V

12.14. Health Professional Consent Form

Health Professional Consent Form v1.0.d

12.15. Interview Schedules

Interview schedules V 1.0.docx

12.16. Interview Schedule – easy read version

Interview schedule easy read V 1.0.docx

12.17. Seizure diary

Seizure diary V1.0.docx

12.18. Carer Invitation Letter

Carers invitation letter - V1.0.docx

12.19. Carer/Consultee Information Sheet

Carer Information Sheet V1.0.docx

12.20. Participant Invitation Letter

Participant Invitation letter V1.0.docx

12.21. Participant Information Sheet

Participant Information Sheet V2.0.docx

12.22. Participant Consent Form

Participant consent form V1.0.docx

12.23. Carer/Consultee Declaration Form

Consultee declaration form V1.0.docx

12.24. GP Information Letter

GP letter V1.0.docx

12.25. Timetable

NIHR Research for Patient Benefit
Title: A randomised controlled feasibility trial of the Book Beyond Words intervention to improve the management of epilepsy in people with learning disabilities

Project/Task	Plan Section	Lead
**Trial Steering Group (TSG)**		
TSG progress and planning meeting		BG
Reference Group meeting		BG
Project meeting		BG
**Phase 1- Trial set-up**	1	
Recruitment of researcher	1.1	MD
Protocol completed	1.2	BG, MD
Application to CLRN support and Portfolio	1.3	MD
BBW training	1.4	MD
**Phase 2 - Recruitment and randomisation**	2	
Identification of eligible patients, consent	2.1	AZ, GP
Randomisation, baseline measures taken	2.2	CRTU, MD
6-month recruitment period	2.3	AZ, MD
**Phase 3 - quantitative and qualitative assessments**	3	
follow-up questionnaires (at 4, 12 and 20 weeks)	3.2	MD
Qualitative interviews	3.3	MD
**Phase 4 - Data analysis, write-up and dissemination**	4	
Quantitative Data analysis	4.1	DW, GB
Qualitative data analysis	4.2	MD
Writeup	4.3	DW, BG, MD, HR
Conferences	4.4	BG
Publications	4.5	BG, MD
Application for funding full-scale trial	4.6	DW, BG, MD, HR

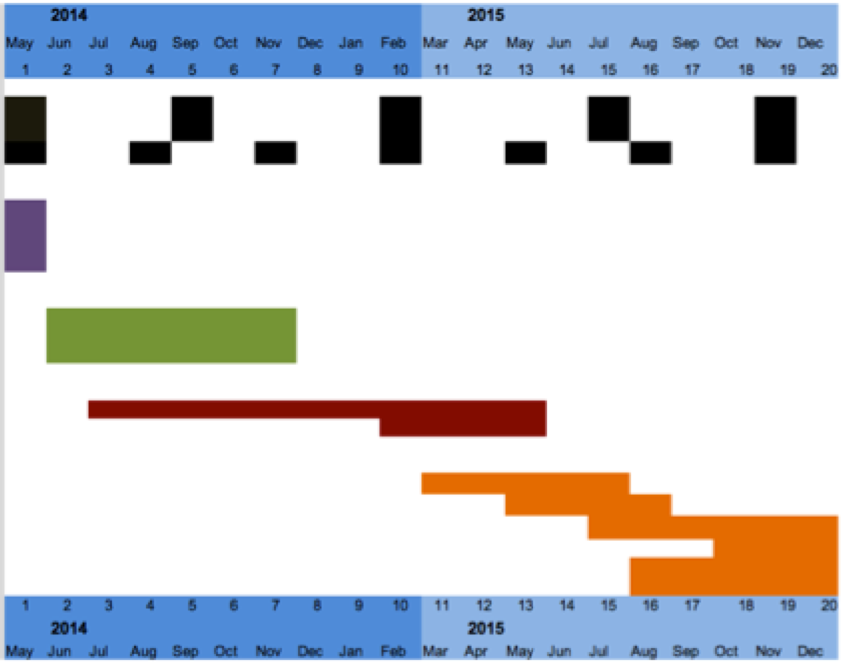

Personnel

Prof Bob Gates BG
Dr Marie-Anne Durand MD
Dr Asif Zia AZ
Dr David Wellsted DW
Dr Georgina Parkes GP
Dr Howard Ring HR
Dr Karin Friedli KF
Dr Garry Barton GB
University of East Anglia CRTU CRTU
